# Identification of hepta-histidine as a candidate drug for Huntington’s disease by *in silico-in vitro- in vivo*-integrated screens of chemical libraries

**DOI:** 10.1038/srep33861

**Published:** 2016-09-22

**Authors:** Tomomi Imamura, Kyota Fujita, Kazuhiko Tagawa, Teikichi Ikura, Xigui Chen, Hidenori Homma, Takuya Tamura, Ying Mao, Juliana Bosso Taniguchi, Kazumi Motoki, Makoto Nakabayashi, Nobutoshi Ito, Kazunori Yamada, Kentaro Tomii, Hideyuki Okano, Julia Kaye, Steven Finkbeiner, Hitoshi Okazawa

**Affiliations:** 1 Department of Neuropathology, Medical Research Institute, Tokyo Medical and Dental University, 1-5-45 Yushima, Bunkyo-ku, Tokyo 113-8510, Japan; 2 Department of Structural Biology, Medical Research Institute, Tokyo Medical and Dental University, 1-5-45 Yushima, Bunkyo-ku, Tokyo 113-8510, Japan; 3 Graduate School of Information Sciences, Tohoku University, 6-3-09, Aramaki-Aza-Aoba, Aoba-ku, Sendai 980-8579, Japan; 4 Cellular System Analysis Team, Computational Biology Research Center, National Institute of Advanced Industrial Science and Technology, 2-4-7, Aomi, Koto-ku, Tokyo 135-0064, Japan; 5 Keio University School of Medicine, 35 Shinanomachi, Shinjuku-ku, Tokyo 112-0012, Japan; 6 Taube/Koret Center for Neurodegenerative Disease, Gladstone Institute of Neurological Disease, and the Departments of Neurology and Physiology, University of California San Francisco, San Francisco CA 94158, USA; 7 Center for Brain Integration Research, Tokyo Medical and Dental University, 1-5-45 Yushima, Bunkyo-ku, Tokyo 113-8510, Japan

## Abstract

We identified drug seeds for treating Huntington’s disease (HD) by combining *in vitro* single molecule fluorescence spectroscopy, *in silico* molecular docking simulations, and *in vivo* fly and mouse HD models to screen for inhibitors of abnormal interactions between mutant Htt and physiological Ku70, an essential DNA damage repair protein in neurons whose function is known to be impaired by mutant Htt. From 19,468 and 3,010,321 chemicals in actual and virtual libraries, fifty-six chemicals were selected from combined *in vitro-in silico* screens; six of these were further confirmed to have an *in vivo* effect on lifespan in a fly HD model, and two chemicals exerted an *in vivo* effect on the lifespan, body weight and motor function in a mouse HD model. Two oligopeptides, hepta-histidine (7H) and Angiotensin III, rescued the morphological abnormalities of primary neurons differentiated from iPS cells of human HD patients. For these selected drug seeds, we proposed a possible common structure. Unexpectedly, the selected chemicals enhanced rather than inhibited Htt aggregation, as indicated by dynamic light scattering analysis. Taken together, these integrated screens revealed a new pathway for the molecular targeted therapy of HD.

Huntington’s disease (HD) is an autosomal dominant disease linked to a CAG repeat expansion in the first exon of the huntingtin gene located in chromosome 4 at position 16.3. A great deal of knowledge has accumulated regarding the pathological mechanisms and physiological functions of the huntingtin (Htt) gene, RNA and protein. The hairpin and other higher structures formed by the CAG repeat sequence of the Htt gene DNA^1^,^2^,^3^,^4^ might affect transcription and DNA repair and subsequently induce instability of the triplet repeat^5^,^6^ and the RNA toxicity^7^ associated with ataxin-3^8^ or non-coding triplet repeat diseases, such as myotonic dystrophy^9^. Moreover, the effect of ataxin-8^10^ might be mediated by CAG siRNA/miRNA^7^ or by sequestration of RNA splicing proteins, such as muscleblind-like 1^11^,^12^,^13^, numerous proteins that interact with Htt, such as HAP1^14^, HIP1^15^, p53^16^, PACSIN1^17^ and other factors. In addition, PQBP1, PQBP3, PQBP5^18^,^19^,^20^ and TERA/p97/VCP^18^,^21^ interact with polyglutamine (polyQ) disease proteins via polyQ tract sequences, including Htt^22^.

However, a combined study of multiple pathological molecules and/or mechanisms has not been performed systematically, and the relative significance of any single mediator molecule among a number of players has been difficult to evaluate. Furthermore, the relative contribution of the molecule to the total pathology remains unclear. Thus, the most essential focus for therapeutic development has not been determined, and no definitive effective therapy against HD has been identified thus far.

The use of the same mouse model enables a comparison of the multiple results from different laboratories on the same platform. For instance, the R6/2 transgenic mouse expressing Htt exon1-Q120 ± 5^23^ has a long history of use in HD research, although whether overexpression of a partial fragment of the mutant protein completely reflects human pathology has been debated. However, it is generally accepted that some features of the R6/2 mouse pathology mimic human HD. We used this model to investigate the pathological function of Ku70^24^; protein-protein interaction screenings identified this protein as molecule that directly interacts with Htt^25^. Furthermore, transgenic overexpression of Ku70 resulted in one of the longest lifespan extensions in R6/2 mice^24^.

In the same study, we found that mutant Htt interacts with Ku70 and impairs its function in non-homologous end-joining (NHEJ), a type of DNA double-strand break repair (DDBR) that functions in non-dividing cells such as differentiated neurons^24^. Mutant Htt expression induces DNA damage 3–4 days prior to the cell death of primary cortical neurons^24^ and activates DNA damage signaling molecules, such as Chk1/2^26^; these changes support the hypothesis that impairment of Ku70 by a mutant protein is an upstream event of neurodegeneration.

Given that previous results have suggested the importance of Ku70 relative to various mediator molecules, we screened chemicals that could inhibit the interaction between Ku70 and mutant Htt. We combined an *in vitro* screen, *in silico* screen, *Drosophila* screen and mouse screen, and we then validated the effect with human iPS cells obtained from HD patients. Consequently, we obtained 6 chemicals that were effective in a *Drosophila* model. We tested 3 of these chemicals and confirmed the therapeutic effect of 2 chemicals in ameliorating the body weight loss and lifespan shortening of R6/2 mice. One chemical was difficult to synthesize on a large scale.

Unexpectedly, hepta-histidine (7H) was the most effective among the chemicals identified from non-biased screens of chemical libraries in this study. We included 7H not only because it was proposed as a candidate on the basis of the *in silico* screening using Discovery Studio but also because it is a polar oligopeptide that might interact with a polyQ tract of Htt via multiple hydrogen bonds of the polar zipper structure, as proposed by the Nobel Prize Laureate Max Perutz^27^. Histidine has a positively charged imidazole group, a positively charged amino group and a negatively changed carboxyl group, and amino acids possessing such polar groups generally contribute to the formation and maintenance of higher protein structures.

L-histidine is a semi-essential amino acid specifically required by young people to grow and repair tissues, and it plays important roles in immunity, gastric secretion, and sexual function. It is metabolized into a neurotransmitter histamine and is necessary for myelin sheath development. The FDA permits the addition of L-histidine to food. Regarding its toxicity, the LD50 of L-histidine is greater than 15,000 mg/kg for oral intake, greater than 8,000 mg/kg for peritoneal injection, and greater than 2,000 mg/kg for intravenous injection in a rat or mouse (pubchem.ncbi.nlm.nih.gov/compound/L-histidine), which is far lower than the effective dose for HD models determined in this study. Therefore, 7H might be a drug seed that can be used safely for HD.

## Results

### First wet screening using single molecule fluorescence spectroscopy

**Figure 1:**
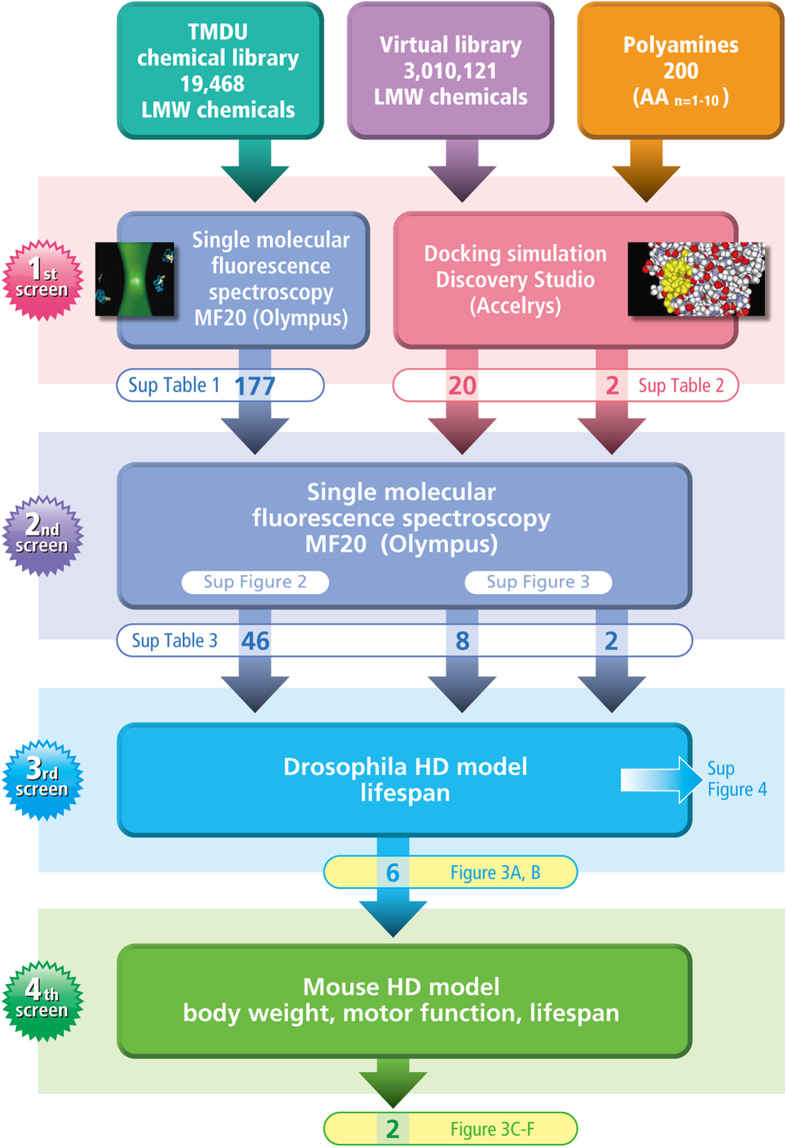
Multidisciplinary screens for drug seeds of Huntington’s disease. The screening strategy to obtain drug seeds for Huntington’s disease is shown schematically. TMDU chemical library, virtual library and polyamines are objectives of the screens. The first screens are based on the ability of a chemical to prevent the interaction between Ku70 and mutant Htt, that is actually confirmed by single molecular fluorescence spectroscopy (MF20) or expected from virtual docking simulation by Discovery Studio. The number of chemicals selected for each screen and the related Supplementary Tables are also shown. MF20 is used for 2^nd^ screen. Drosophila and mouse model (R6/2 mice) are used for 3^rd^ and 4^th^ screens. Selected chemicals are listed in the indicated Figures and Supplementary Figures.

We employed both wet and dry screens (MF20 and Discovery Studio, respectively) for the initial step, performed a secondary screening with single molecule fluorescence spectroscopy, and examined the phenotypic effects in *Drosophila* and mouse models as the third and fourth screens (Fig. 1).

For the first wet screening of our in-house chemical library (19,468 chemicals or peptides) at the TMDU Chemical Biology Screening Center (http://mechpc5.tmd.ac.jp:3000/cbdb), we employed single molecule fluorescence spectroscopy (MF20, Olympus) and examined the effect of chemicals or peptides from the library on the interaction between normal Ku70 protein (Ku70-HisTag expressed in *E. coli* by the pET28 vector) and mutant Htt protein (GST-HttExon1-110Q). This apparatus detects the interaction of a fluorescent molecule and a non-fluorescent molecule in 1 × 10^−15^ L in several seconds and is available for high-throughput screening (Supplementary Figure 1a).

Prior to the start of the screening, we tested the efficiency of two methods, fluorescence correlation spectroscopy (FCS) and fluorescence intensity distribution analysis-polarization (FIDA-PO). The former was used to evaluate the diffusion time and the latter was used to estimate the fluorescence polarization of a fluorescent molecule (Supplementary Figure 1b). In both cases, when a fluorescent protein binds to another non-fluorescent protein, the diffusion time and the polarization both increase (Supplementary Figure 1b). Indeed, we fluorescently labeled and resolved the Ku70-HisTag protein at 5 nM in a volume of 40 μl. Next, different concentrations of anti-Ku antibody, as a positive control, or GST-HttExon1-110Q were added to the solution. Compared to their respective negative controls (i.e., non-specific mouse IgG and GST protein), FIDA-PO but not FCS detected a change due to the positive control and a change caused by GST-HttExon1-110Q at 500 nM (Supplementary Figure 1c). Thus, we selected FIDA-PO in this condition (5 nM Ku70-HisTag and 500 nM GST-HttExon1-110Q) for further screening.

Next, we performed the first screening using our in-house chemical library (19,468 chemicals or peptides). Fluorescence polarization was recorded 10 times/well in two wells using a single chemical from the library (Supplementary Figure 1d). In the absence of GST-HttExon1-110Q in the solution, the fluorescence polarization score was approximately 120, which indicates the non-binding state of Ku70. In the presence of GST-HttExon1-110Q, the fluorescence polarization score increased to nearly 140. With the addition of a molecule from the chemical library, the score was decreased, increased or remained unchanged. A decreased score reflected the dissociation of Ku70-HisTag from GST-HttExon1-110Q by the molecule. An increased score could not be explained well, but it might be due to either the polymer or aggregate formation of Ku70-HisTag in the presence of the chemical or the high polarization of the chemical itself. An unchanged score meant no effect of the molecule on interaction between Ku70-HisTag and GST-HttExon1-110Q.

It was possible for intermolecular binding to occur with Ku70-HisTag. However, our data indicated that the intermolecular binding of Ku70-HisTag was not as strong as the interaction between Ku70-HisTag and GST-HttExon1-110Q (Supplementary Figure 1d).

We used 384 wells in a glass-bottom plate for screening; 2 wells were used for the same chemical. Internal positive and negative controls were added at four positions, including the wells in the first and last positions (Supplementary Figure 1d). Eventually, for the second screening, we selected 177 chemicals whose score decreased to values that were between +5% and −5% of the mean value (Supplementary Table 1).

The Ku70-HisTag expressed and purified from *E. coli* was confirmed to be in a native functional state by pull-down assay with nuclear extract of HEK293 cells showing that Ku70-HisTag possessed the ability to interact with endogenous Ku80 (Supplementary Figure 1e). In this experiment, Ku80 was not pulled down with Tau-HisTag as a negative control (Supplementary Figure 1e).

### First dry screening using Discovery Studio

Next, we performed a virtual screening of chemicals using the docking simulation software Discovery Studio. We retrieved the structure of Ku70 (ID: PD0220) from the Protein Data Bank (PDB) (http://www.rcsb.org/pdb/explore/explore.do?structureid=1JEY) and screened 3,010,121 chemicals in Chemical Available Purchase (CAP) 2006 (Dassault Systems BIOVIA Cooperation, San Diego) using LibDock.

**Figure 2:**
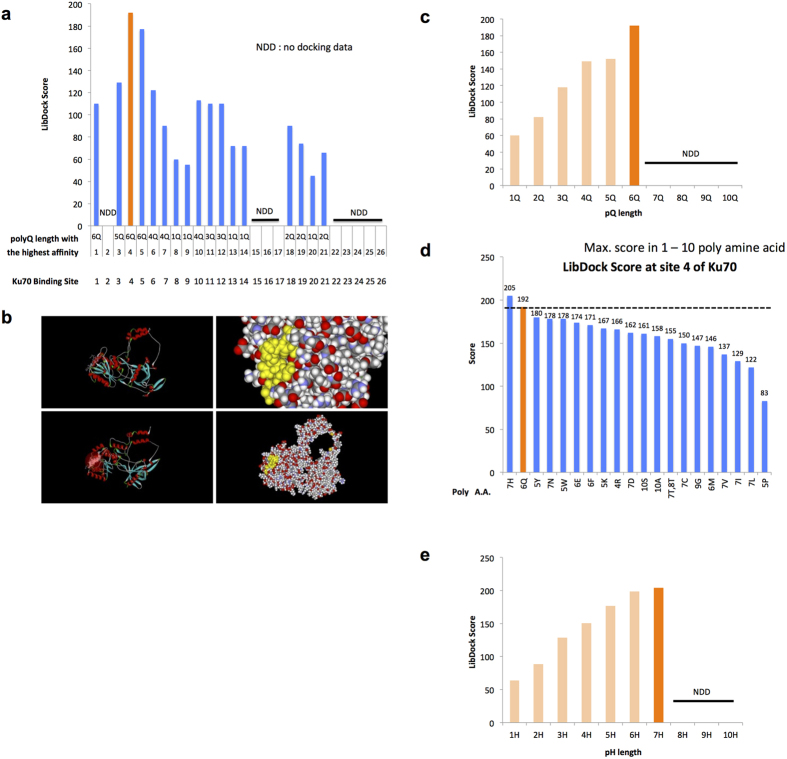
Docking simulation of poly(amino acids) to Ku70. (**a**) Probabilities of interaction between polyglutamine peptides and 26 potential surfaces of Ku70 are shown as LibDock scores expected by Discovery Studio. (**b**) Image showing the simulation of the interaction of hexa-glutamine (6Q) at site 4 of Ku70 expected by Discovery Studio. (**c**) LibDock scores (scores for expected binding affinity) of various lengths of polyglutamine peptides in a binding simulation with site 4. (**d**) The 20 polypeptides with the highest LibDock scores for binding to site 4. 7H had a higher score than did polyglutamine of various lengths (polyQs). (**e**) LibDock scores of various lengths of polyhistidine peptides for simulations of binding at site 4.

First, we performed a docking simulation of various lengths of polyQ to various segments of Ku70 (Fig. 2a, site 1–26) and revealed binding of the polyQ peptide to a dip in the N-terminal region (site 4), which had been previously shown to interact with mutant HttExon1^24^. A 3D image of the docking of 6Q to the dip was generated using Discovery Studio (Fig. 2b). Interestingly, 6Q (Gln-Gln-Gln-Gln-Gln-Gln) bound to site 4 with the highest affinity, and a shorter polyQ bound with a lower affinity (Fig. 2c). Longer polyQs (length of at least 7) were not expected to bind to site 4, which might be due to the limitation of the algorithm in the software.

Thus, site 4 was considered the most plausible binding site for Htt, and we screened 3,010,121 chemicals for an interaction with this site. We ranked the chemicals from highest to lowest based on the docking score (LibDock score) and selected the top 20 chemicals that were commercially available for the second screening with MF20.

Interestingly, the software predicted that 7H (His-His-His-His-His-His-His) could bind to Ku70 at site 4 with higher affinity than 6Q (Fig. 2d) when we added various lengths of all types of polyamines (lengths of 1–10 a.a.) to the *in silico* screening. Of the various lengths of poly-His, 7H was predicted to interact with Ku70 with the highest affinity (Fig. 2e). Thus, we included 6Q and 7H for the second screening.

### Second screening using single molecule fluorescence spectroscopy

In total, 177 chemicals from the first screening with MF20, 20 chemicals from the docking simulation by Discovery Studio, and 2 polyamines (6Q, 7H) were examined in the second screening with MF20 at multiple concentrations (Supplementary Figures 2, 3). The second biochemical screening confirmed that 46, 8 and 2 molecules, respectively, inhibit the interaction between mutant Htt and Ku70 (Fig. 1, Supplementary Table 3).

In this process, we unexpectedly observed that the positive ratios in the second screening were higher for the chemicals initially screened by Discovery Studio and for the assumption-based polyamines than the positive ratio from the first screening of the chemical library by MF20. These chemicals were subsequently examined in the third and fourth screenings using animal models.

### Third phenotypic screening using an HD fly model

Because it was impossible to synthesize three of the 59 chemicals at a large scale, which was essential for animal models, we examined 56 chemicals in the third biological screening using an HD fly model that had been previously developed in our laboratory^28^,^29^. The fly model expresses the Htt protein in motor neurons under the control of OK6 motoneuron-specific driver, and lifespan can be used as a phenotypic marker in chemical or genetic screening^28^,^29^.

For instance, we synthesized 7Q and 12Q in addition to 6Q for references. The amount of 7Q recovered from the synthesizer was sufficient for the *Drosophila* screen, whereas we were unsuccessful at synthesizing a sufficient amount for the mouse model. The synthesis of 12Q was completely unsuccessful in terms of producing the necessary quantity for a *Drosophila* model. The tendency of 7Q and 12Q to aggregate at a high concentration during the synthesis may account for the failures observed in the chemical synthesis. However, 7H was successfully synthesized.

**Figure 3:**
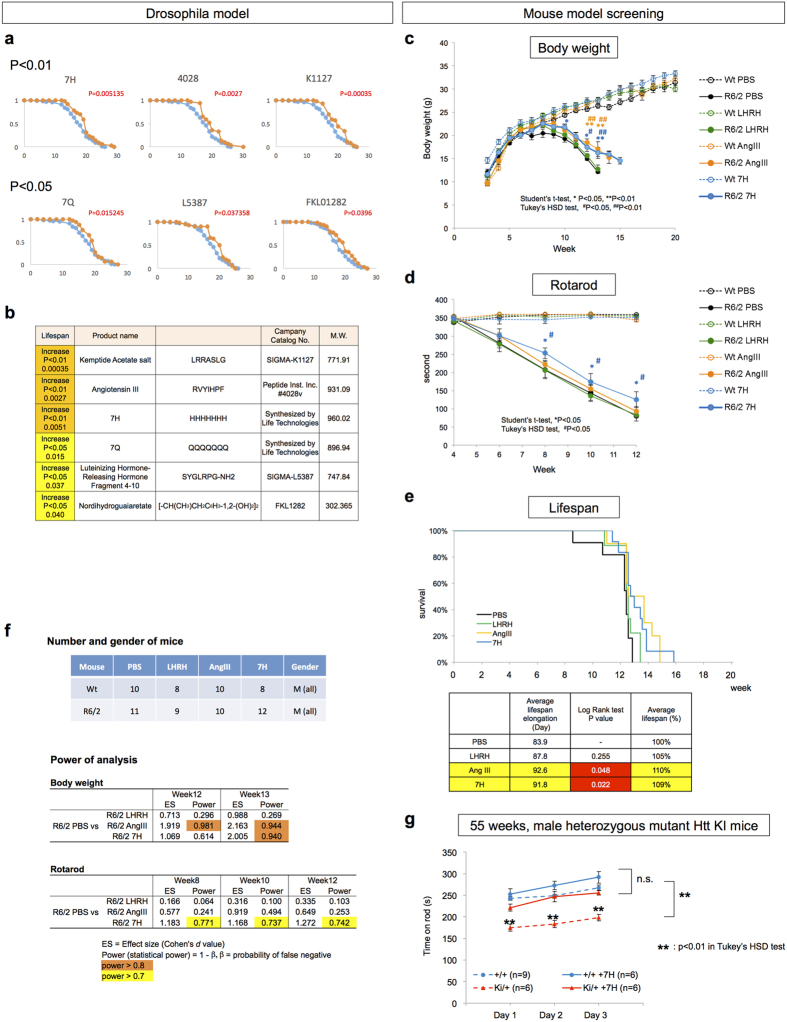
Third and fourth screenings with Drosophila and mouse HD models. (**a**) Six candidate chemicals were selected in the third screening of the effect on the lifespan of the *Drosophila* HD model (Gal4-UAS model overexpressing human mutant Htt Exon1-103Q in motor neurons by OK6 driver^49^). The chemicals were fed in corn meal medium at a final concentration of 500 μM. For the control medium, only solvent was added. Twenty virgin female flies were maintained per vial and the number of flies was counted every 2–3 days when the meal was replaced with a new one. Log-rank test was used for statistics. (**b**) Information about the selected low molecular weight (LMW) molecules. (**c**) The fourth screening was performed with background (WT) and R6/2 mice fed with the candidate chemicals from the third screening. Chronological changes of body weight are shown as mean +/− S.E. *p < 0.05 and **p < 0.01 in Student’s t-test. ^#^p < 0.05 ^##^p < 0.01 in Tukey’s HSD test. (**d**) Motor function of background (WT) and R6/2 mice fed with the candidate chemicals was analyzed by Rotarod test. Mean +/− S.E. are shown in graph. (**e**) Lifespan of background (WT) and R6/2 mice fed with the candidate chemicals. The results of the log-rank test are shown in the lower table. (**f**) The upper list indicates number and gender of mice used for each chemical and control (PBS) in Fig. 2c–e. The lower lists indicate the power of statistics at the time-points of each analysis where we judged statistically different. (**g**) The effect of 7H on the motor function of mutant Htt-KI mice was analyzed by Rotarod test at 55 weeks of age. The three peptides were peritoneally injected for 1 week before the analysis. Rotarod stay times on day 1–3 were compared among background mice (+/+), back ground mice injected with 7H (+/+ +7H), heterozygous mutant Htt knock-in mice (KI/+), and mutant Htt heterozygous knock-in mice injected with 7H (KI/+ +7H). Tukey’s HSD test was used for statistics.

After overcoming such technical issues, we examined 56 chemicals for the third screening and mixed these chemicals with food at 500 μM (Supplementary Figure 4). The third screening revealed that 3 chemicals (7H, #4028, and K1127) extended the lifespan, as indicated by a log-rank test at p < 0.01, and an additional 3 chemicals (7Q, L5387, FKL01282) exerted an effect with p < 0.05 (Fig. 3a,b). Although the lifespan extensions were not large, the effects were reproducible.

### Fourth phenotypic screening using Htt-exon1-transgenic mouse model

Thus, we proceeded to the fourth screening with R6/2 transgenic mice expressing human mutant Htt exon 1, a well-known mouse model with the earliest onset, shortest lifespan, and most severe symptoms relative to those in various HD mouse models. Despite the debate about whether overexpression of Htt exon 1 might produce some artifacts in the pathology or symptoms of HD, compared to other mutant Htt-KI, -YAC-Tg or -BAC-Tg mice, R6/2 is clearly the most efficient model for screening multiple chemicals in the shortest duration^30^.

In the fourth screening with mice, we injected these candidate chemicals intraperitoneally at 50 μg/g body weight. We selected this concentration because LH-RH is used at 2 mg/kg for rat without showing the toxicity according to information from Mitsubishi Tanabe Pharma Co., Ltd. (https://medical.mt-pharma.co.jp/di/file/if/f_lhr.pdf). Angiotensin II is used at 1mg/kg/day^31^ and Angiotensin was used at 1–30 mg/kg/day^32^ for peritoneal injection. Considering with the concentration, we increased the amount at one order, confirmed that the dose did not induce acute toxicity, and employed 50 mg/kg (=50 ug/g) for the experiment.

With regard to body weight changes, R6/2 mice showed a decline at approximately 9 weeks of age (Fig. 3c). This decline continued until death but it was reduced by Angiotensin III (#4028, RVYIHPF) and 7H (p < 0.01 or p < 0.05 at each weak of age). Motor function, as evaluated using a rotarod test, was also improved by 7H, but was not obviously improved by Angiotensin III (Fig. 3d). Angiotensin III and 7H also prolonged the lifespan in the most severe HD model, R6/2 mice (p < 0.05). The effect of LH-RH 4–10 peptide (L5387) was not confirmed in the three tests (Fig. 3e,f). Although we observed that the lifespan of the HD fly model was prolonged by 7Q (Fig. 3a,b), due to the aggregation tendency of 7Q during synthesis, we could not obtain a sufficient amount of 7Q for application in the fourth screening using the HD mouse model.

Consistent with the recovery of R6/2 mice, we found a rescue effect of Angiotensin III, LH-RH 4–10 peptide and 7H on the DNA damage of the striatal neurons of R6/2 mice using immunohistochemistry with anti-γH2AX and anti-53BP1 antibodies (Supplementary Figure 5a,b). However, none of the three chemicals decreased the frequency of inclusion bodies of mutant Htt in the striatum of R6/2 mice when we stained the striatum with anti-Htt antibody (EM48) (Supplementary Figure 5c). Western blot analyses using anti-γH2AX, anti-53BP1 and anti-Htt antibodies also supported both the amelioration of DNA damage by 7H, #4028 (Angiotensin III) and L5387 (LH-RH 4–10 peptide fragment) and the insensitivity of the mutant Htt aggregation to these chemicals in the striatum of R6/2 mice (Supplementary Figure 5d). Ubiquitination of abnormal proteins was also not affected by these chemicals (Supplementary Figure 5e). Taken together, these results support the hypothesis that the three chemicals restored the phenotypes of the HD fly and mouse models by inhibiting the interaction between the disease protein and the target physiological molecule but not by suppressing aggregation, as we expected at the beginning of this screening study. However, since we employed only a single dose for mouse experiment, we need to select appropriate doses of these peptides in the future.

K1127 (Kemptide acetate salt) was not employed for the fourth screening because a computer search suggested a possible similarity between Kemptide acetate salt and glatiramer acetate (Copaxone), which had been tested in an HD mouse model (N171-82Q transgenic mice) and has been previously reported to ameliorate the metabolic activity and parameters of motor function, including jumping counts and stereotypic counts^33^. However, a detailed search ruled out a direct relationship between Kemptide acetate salt and these results. Thus, Kemptide acetate salt may be the next target molecule for evaluation.

### Validation of phenotypic recovery with Htt-KI mice

Regarding the small size effects of Angiotensin III, LH-RH 4–10 peptide fragment and 7H on phenotypes of R6/2 mice, we suspected the small effect could be due to too severe phenotypes of R6/2 mice and asked whether the effect might become more visible in the other HD model expressing full-length Htt at a physiological level. Therefore we tested the effect of 7H on the motor function of mutant Htt-KI mice (Hdh^*Q111*^ mice)^34^ at the age of 55 weeks around the onset (Fig. 3g). We found the definitely positive therapeutic effect of 7H on motor function of Htt-KI mice. However also in this case the extent of recovery was small because the difference of motor function between Htt-KI and the control sibling mice was not large at this timepoint. Further investigations with more aged Htt-KI mice are planned and will be reported elsewhere.

### Common molecular structure of candidate chemicals

**Figure 4:**
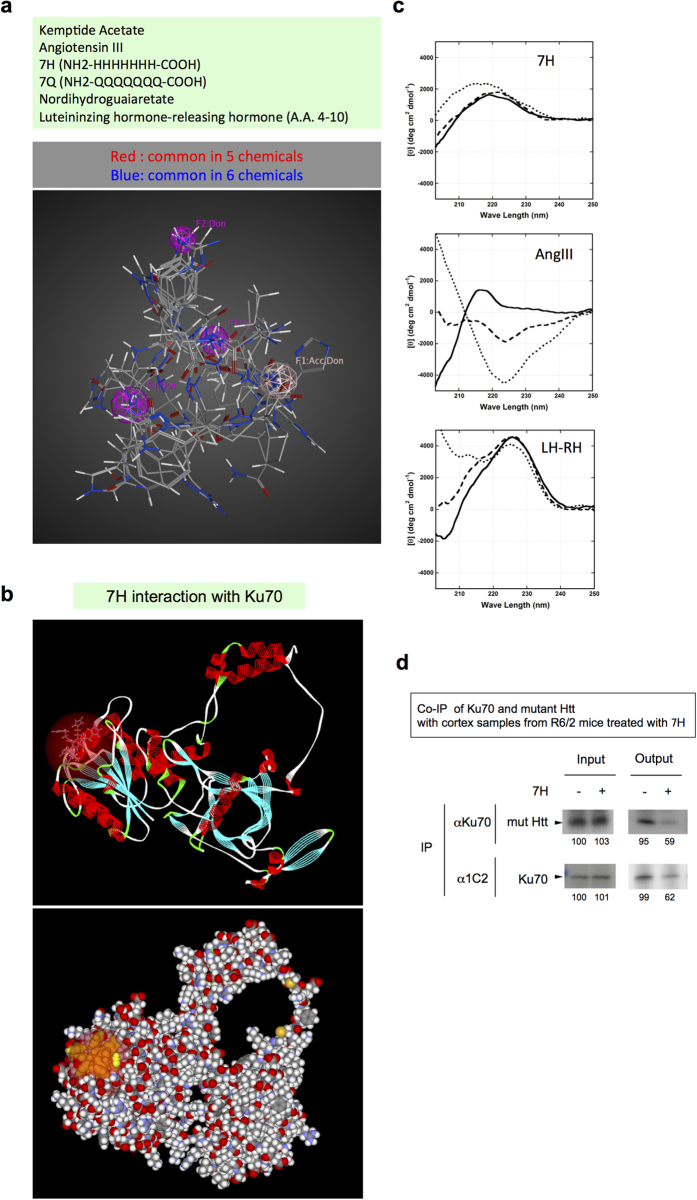
Expected common structure of candidate chemicals. (**a**) The structures of six chemicals that were effective for the *Drosophila* HD model are merged to extract the common features of the ternary structure. F1 is a donor/acceptor for hydrogen bonds and F2-4 are acceptors for hydrogen bonds. The positions for the hydrogen bond are common. The covalent bonds are colored red if the position is shared among five chemicals and are colored blue if the position is shared among the six chemicals. (**b**) The incorporation of 7H at the dip in the N-terminal region of Ku70 was visualized using Discovery Studio. (**c**) CD spectra of the three peptides, 7H, AngIII and LH-RH 4–10 partial peptide, are shown. In each panel, solid line, dashed line, and dotted line stand for 0, 10, and 20% HFIP, respectively. (**d**) Co-IP of Ku70 and mutant Htt with cerebral cortex samples from R6/2 treated with 7H. The results obviously show decreased interaction between Ku70 and mutant Htt after treatment of 7H.

We attempted to identify the common molecular structure of the selected chemicals. By merging the six chemicals, we determined a common structure (Fig. 4a). F1 functions as a donor/acceptor in the hydrogen bond, and F2-4 function as acceptors in the hydrogen bond. The structure consists of four hydrogen bond acceptor/donor sites, and it is considered essential for candidate chemicals. Interestingly, 6Q and 7H share a similar structure. This result suggested bi-directional binding of 7H and other candidate chemicals to Ku70 and polyQ sequences. Discovery Studio predicted that the binding affinity (LibDock score) of 7H to the dip of Ku70 would be higher than that of 6Q (Fig. 2d). The incorporation of 7H at the dip in N-terminal region of Ku70 was also visualized using Discovery Studio (Fig. 4b).

On the other hand, amino acid compositions of 7H, AngIII, and LH-RH 4–10 peptide fragment were very different though they showed similar biological functions. To explain the discrepancy, we assumed that they might take similar conformations in the binding pocket of the target protein. To test the hypothesis, we investigated their secondary structure under the hydrophobic condition to know conformations of the 3 peptides in the bound states, which mimicked the binding pocket because it had higher hydrophobicity than aqueous solution.

We measured the far-UV circular dichroism (CD) spectra of the peptides at 25 °C in PBS containing 0, 10 or 20% 1,1,1,3,3,3-hexafluoro-2-propanol (HFIP), which induces a typical hydrophobic environment, by using Jasco J-720 spectropolarimeter (Fig. 4c). At 0% HFIP, the CD spectra of the 3 peptides overall showed a common feature; there was a positive band around 220 nm, and according as the wavelength decreased, the ellipticity decreased to large negative value (Fig. 4c). This feature suggests the random coil conformation, implying that all the 3 different peptides commonly take random coil conformation before binding to the protein.

When the concentration of HFIP was increased, the ellipticity of the 3 peptides commonly changed from negative value to positive one around 200 nm, suggesting that they took the turn-like conformation. Furthermore, a negative band, which usually suggests helical conformation, was observed around 220 nm in the CD spectra of AngIII (Fig. 4c). In this case, however, the negative value suggested a couple of turns rather than a helical conformation but just, because AngIII is composed of only 7 amino acid residues. Together, the conformations of the 3 peptides were suggested to take turn-like conformations in the binding pocket under the hydrophobic condition.

Consistently, we obtained the similar results from *in silico* screening. As shown in Fig. 4b the conformation of 7H was turn-like in the binding pocket of Ku70 and the conformations of AngIII and LH-RH 4–10 peptides were basically similar (data not shown). These results form our wet and dry experiments supported our hypothesis that the three peptides take the similar structure in the binding pocket of the target protein.

We also tested whether 7H could inhibit the physical interaction between mutant Htt and Ku70 *in vivo*, by co-immunoprecipitation assay using the cortex tissues of R6/2 mice treated with 7H (Fig. 4d). The results indicated that 7H obviously inhibited interaction between mutant Htt and Ku70 *in vivo* (Fig. 4d).

Taken together, these results suggested a role of 7H as a competitive antagonist of polyQ in the interaction with Ku70. Presumably, 7H competitively binds to the dip of Ku70 with polyQ protein and prevents the interaction between Ku70 and mutant Htt.

### Effect of candidate chemicals on Htt aggregation

**Figure 5:**
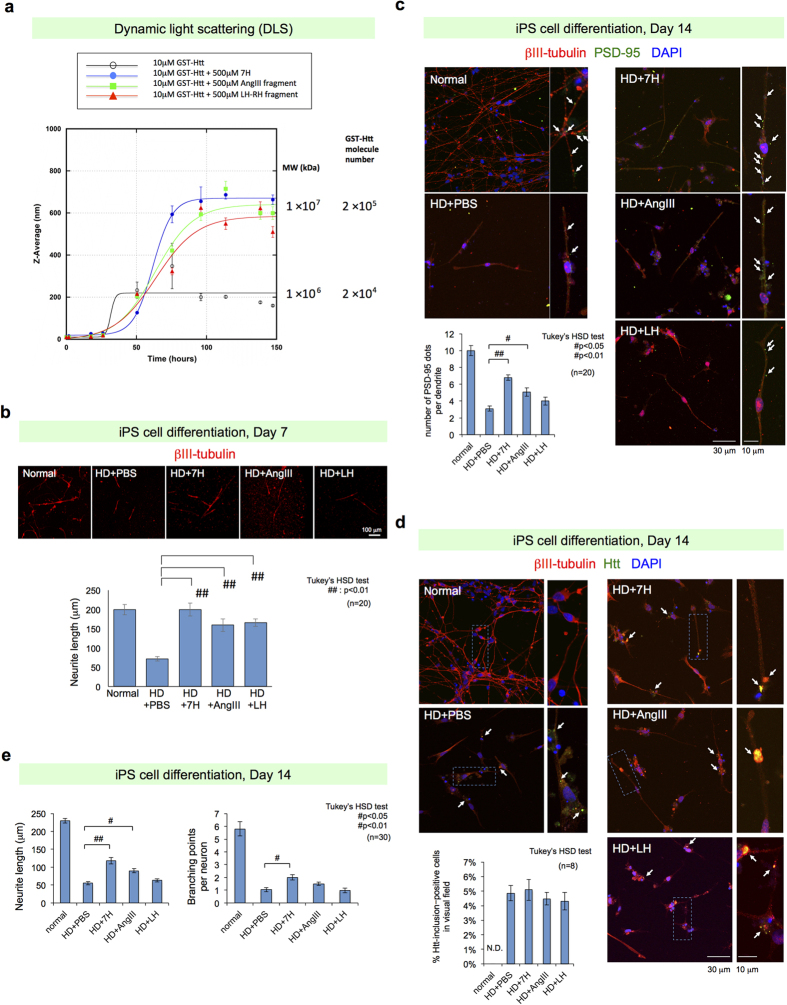
Effect of candidate chemicals on Htt aggregation and human neurons from iPS cells of an HD patient. (**a**) The final three candidate chemicals, i.e., 7H, #4028 (Angiotensin III), and L5387 (LH-RH 4-10 peptide fragment), affect the dynamic light scattering (DLS) measurements of GST-HttExon1-110Q aggregation *in vitro*. The three chemicals noticeably increased the molecular weight of the final aggregation products, whereas they slowed the initial phase of aggregate formation. (**b**) Two chemicals that were effective in the mouse HD model were re-examined using human neurons differentiated from the iPS cells of the HD patient. The decreased neurite length was reversed by treatment with 7H and Angiotensin III. Regarding the neurite length, we measured neurite lengths of two randomly selected neurons in a well, and the data of 10 wells were integrated. Mean +/− S.E. are shown in graph. ^##^p < 0.01 in Tukey’s HSD test. (**C**) Spine formation was evaluated using immunohistochemistry for PSD95. 7H and the Angiotensin III fragment reversed the decrease in the PSD95 spot number. PSD number per neurite was measured in two randomly selected neurons in a well and divided per neurite length (μm). The values from multiple wells (n = 10) were integrated to mean +/− S.E. of each treatment in the graph. ^#^p < 0.05 and ^##^p < 0.01 in Tukey’s HSD test. (**d**) Mutant protein aggregation was evaluated using immunohistochemistry for Htt. No effect was observed using the three chemicals. The number of neurons with Htt inclusion bodies was calculated in a randomly selected visual field of each well. and the mean +/− S.E. of 8 wells were calculated. (**e**) The decreased neurite length and branching point number of HD neurons were reversed by treatment with 7H and #4028 (Angiotensin III) at day 14 after neural differentiation. The branching number of neurite per a neuron was calculated for 3 or 4 randomly selected neurons in each well, and the mean +/− S.E. of 8 wells (30 neurons) were calculated. Statistical analyses used for (**a–e)** are shown in the figures.

Furthermore, candidate chemicals might interact with polyQ sequences. We investigated the effect of candidate chemicals on Htt aggregation using *in vitro* and *in vivo* methods. Dynamic light scattering (DLS), a physics technique for measuring molecular size and the corresponding molecular weight in solution^35^, was employed to analyze the effect of candidate chemicals on the dynamic process of GST-HttExon1-110Q aggregation *in vitro*. This technique successfully measured the dynamics of Htt aggregation^36^. Unexpectedly, we found that the three candidate chemicals, 7H, #4028 (Angiotensin III) and L5387 (LH-RH 4–10 peptide fragment), remarkably increased the molecular weight of the final aggregation products (Fig. 5a). Moreover, during the initial phase of incubation, the process of aggregate formation was slow in the presence of the candidate chemicals but increased rapidly to form larger aggregates (Fig. 5a). On the basis of these findings, we conclude that these chemicals most likely interact with and change the dynamics of mutant Htt. The chemicals prolong the time in which mutant Htt exists as a monomer, shorten the time that GST-Htt exists as a small polymer that is most likely a protofibril, and increase the proportion of large polymers.

Taken together, these results support the hypothesis that the candidate chemicals interact with Htt polyQ protein either to promote aggregation or at the very least, to not prevent aggregation in a long time span.

### Validation using human neurons differentiated from HD iPS cells

Finally, to validate the therapeutic effect on human neurons, the three candidate chemicals, Angiotensin III (#4028, RVYIHPF), 7H (HHHHHHH) and LH-RH 4–10 peptide fragment (L5387, SYGLRPG-NH_2_), were tested on human neurons derived from iPS cells of HD patients who had been established in the Coriell Institute (https://catalog.coriell.org). Before usage, the characteristics of the iPS cells were re-examined. Neurons were differentiated from iPS cells according to a previously described protocol^37^, and these cells contained 80% βIII-tubulin-positive neurons and 5% GFAP-positive glia. We also found that DARPP32-positive cells were hardly detected among βIII-tubulin-positive neurons in our differentiation protocol, and most of them were TBR1-positive neurons corresponding to V–VI cortical layers (Supplementary Figure 6). Thus our object in this experiment was pan-neuron but not medium spiny striatal neurons. Confocal microscopy revealed a therapeutic effect of the screened chemicals such that both the Angiotensin III (#4028, RVYIHPF) and 7H (HHHHHHH) improved the dendritic length at day 7 (Fig. 5b). Immunocytochemistry using PSD95 on day 14 revealed that the spine density was restored by the addition of 7H and the Angiotensin III (Fig. 5c). However, these chemicals did not reduce the number of inclusion body-positive neurons (Fig. 5d), as was expected from DLS results (Fig. 5a). Recovery of dendritic length and branching points was also confirmed at day 14 (Fig. 5e). Taken together, these results confirmed the therapeutic effect of final candidate chemicals on human neurons, which was independent of inclusion body formation.

## Discussion

### Hepta-histidine (7H), a seed for drug development against HD

In this study, we screened candidate drug seeds for the treatment of HD via multi-step screenings targeting the prevention of the abnormal interaction between Ku70 and mutant Htt. Consequently, we identified two definite and additional four promising seeds for drug development against HD. These chemicals shared common structural features and may interact with both Ku70 and mutant Htt. Unexpectedly, we found that 7H was more effective than numerous chemicals identified from a non-biased screening of chemical libraries in this study. Another interesting finding was that the final candidate chemicals did not inhibit but rather promoted the aggregation of mutant Htt. The latter result may provide additional insight about whether polyQ aggregates are toxic, protective or both.

We included 7H not only because it was proposed as a candidate on the basis of the *in silico* screening using Discovery Studio but also because it is a polar oligopeptide that might interact with a polyQ tract of Htt via multiple hydrogen bonds of the polar zipper structure, as proposed by the Nobel Prize Laureate Max Perutz^27^. Histidine is one such polar amino acid that has a positively charged imidazole group, a positively charged amino group and a negatively changed carboxyl group, and amino acids possessing such polar groups generally contribute to the formation and maintenance of higher protein structures.

Oligopeptides are efficiently absorbed from the intestine to blood and are transported from the extracellular fluid into the cytoplasm by membrane transporters, such as ATP-binding cassette transporters (ABC transporters)^38^,^39^ or proton-dependent oligopeptide transporters (PTR)^40^. Thus, the positive results *in vivo* with *Drosophila* and mouse models in this study could be explained by the efficient incorporation of oligopeptides into cells, which is advantageous for other low molecular weight chemicals when these oligopeptides progress to clinical trials.

Moreover, if 1,000 mg of 7H were uniformly diluted in a 70 kg human body, the final concentration is approximately 12 μM, which is within the range of concentrations that inhibit the abnormal interaction between Ku70 and mutant Htt as found in this study. The concentration necessary to inhibit the interaction is one order or magnitude lower (Supplementary Figure 3); thus, if the efficiency from the intestine to blood and that from the blood to brain is high, then an acceptable amount of hepta-histidine, such as 0.1–1.0 g/day (14–140 mg/kg/day), might be employed for a 70 kg person.

Another advantage of hepta-histidine is that oligo-histidine has been previously used as a tag of fusion proteins in a broad range of biological experiments for a long time, and the low toxicity has been previously established from these studies. Although we did not analyze the dose-effect or dose-toxicity relationships of hepta-histidine in this study, it is expected that a relatively high dose would be tolerable in model animals and human patients in a pre-clinical study and clinical trials. Importantly, the Roche license for the experimental usage of the hexa-histidine tag (EP0282042) expired in 1994.

Finally, to exclude the concern that 7H, Angiotensin III and LH-RH 4–10 peptide fragment might suppress the DNA damage repair function of Ku70 as a side effect through their interaction with Ku70, we analyzed the effect of these chemicals on DNA-PK activity with the method described previously^24^ and found that the three peptides did not suppress the DNA-PK activity at 50 and 100 μM (Supplementary Figure 7). Instead, we unexpectedly found that 7H enhances DNA-PK activity (Supplementary Figure 7). Since we have already shown that 7H dissociates abnormal interaction between mutant Htt and Ku70, the result is not contradictory to our conclusion. The result also confirms that 7H and the other peptides have no side effect. It rather suggests that 7H has an additive function to enhance the therapeutic effect on HD.

### Angiotensin III: another type of seed drug

Angiotensin III, RVYIHPF (Arg-Val-Tyr-Ile-His-Pro-Phe), is located at amino acids 35–41 in Angiotensinogen (NCBI reference Sequence: NP_000020.1). Angiotensinogen (435 amino acids) is expressed in the liver and activated in adipocytes and is then cleaved into Angiotensin I (Asp-Arg-Val-Tyr-Ile-His-Pro-Phe-His-Leu) by a protease, renin, in the kidney. Furthermore, Angiotensin I is converted to Angiotensin II (Asp-Arg-Val-Tyr-Ile-His-Pro-Phe) by the removal of two amino acids from the C-terminus by the angiotensin converting enzyme (ACE)^41^. The trimming of one or two amino acids produces Angiotensin III and Angiotensin IV (Val-Tyr-Ile-His-Pro-Phe), respectively. Furthermore, Angiotensin II has the strongest biological activity among the Angiotensins^41^. The vasopressor activity is also high for Angiotensin II but not for Angiotensin I and is low for Angiotensin III (40% of the Angiotensin II activity) and Angiotensin IV^42^. In addition, the mineralocorticoid activity of Angiotensin III is similar to that of Angiotensin II^42^.

Thus, to proceed to clinical translation, it is necessary to decrease the mineralocorticoid activity of Angiotensin III. If the therapeutic activities of Angiotensin IV or other shorter peptides are confirmed against HD pathology in the same screenings, such derivatives of Angiotensin III will also be candidate drugs.

### LH-RH peptide fragment and other chemicals are effective in a fly model

Although the therapeutic effect of the LH-RH 4–10 peptide fragment was not confirmed in the HD mouse model, the effect was confirmed in an HD fly model. Thus, this peptide might still be viable as a candidate drug. In this regard, other chemicals that were effective in fly models should also be re-evaluated in the future.

Specifically, the Kemptide acetate salt (Leu-Arg-Arg-Ala-Ser-Leu-Gly) identified in this study is a strong candidate. It is a substrate of protein kinase A (PKA). Additionally, our finding suggests that the binding of mutant Htt to multiple proteins at this motif for PKA recognition impairs PKA signaling and subsequently results in impaired recognition and decreased memory in HD, as previously reported^43^.

Regarding the blood-brain barrier (BBB), the molecular weights of the chemicals selected from our third screening with *Drosophila* model were all greater than the cut-off value (400–600 Da) for transmembrane diffusion proposed by Lipinski^44^, except FKL01282 (nordihydroguaiaretate, NDGA) (Fig. 3b). Thus, NDGA is another candidate drug seed for HD. Interestingly, another group also reported that NDGA was effective on R6/2 mice^45^. Taken together, the results strengthen the reliability of the chemicals selected from our integrated screening.

Although the molecular weights of the chemicals, including Kemptide acetate, are greater than 500 kDa, peptides and proteins larger than 600 kDa are known to cross the BBB not only by transmembrane diffusion but also by peptide transporter or endocytosis^46^. Indeed, the 7,800 Da cytokine-induced neutrophil chemoattractant-1 (CNC-1) is known to cross the BBB^47^.

## Conclusion

We confirmed the therapeutic effect of 2 oligopeptides against HD pathology using *in vitro* and *in vivo* experiments in a mouse model and with human iPS cells. However, 4 candidates still remain that have not been tested due to technical reasons or due to an effect at the borderline level when they were tested with the most severe HD mouse model. Thus, the six chemicals or their derivative chemicals could be candidate seeds for the development of therapeutic drugs against HD.

## Methods

### Construction of GST-Htt and His-Ku70 vectors

Human huntingtin exon 1 cDNA containing 110 or 20 CAG repeats was subcloned into pGEX-3X (GE Healthcare) as previously described^24^. Mouse Ku70 cDNA was amplified from the RIKEN full-length enriched mouse cDNA library with 5′-AAAGGATCCATGTCAGAGTGGGAGTCCTA-3′ and 5′-AAACTCGAGTGTTCTTCTCCAAGTGTCTGA-3′ primers and subcloned into the BamHI and XhoI sites of pET28(a) (Clontech).

### Protein expression and purification

Plasmids for the GST-Htt and HisTag-Ku70 proteins were transformed into *E. coli* Rosetta (DE3) (Novagen) competent cells. The transformed cells were cultured in shaker at 37 °C and 200 rpm. When the OD at 600 nm reached 0.3, IPTG (final concentration of 1.0 mM) was added and further incubated at 37 °C at 200 rpm for 2 hours. *E. coli* cells were collected by centrifugation and lysed in 20 ml of PBS containing 0.1% Tween 20, 0.1% Lysozyme (Sigma), and 1/500 Protease Inhibitor Cocktail III–EDTA-free (Calbiochem) for the GST-Htt protein and in 20 ml of PBS containing 10 mM imidazole, 1% Triton X-100, and 1/500 Protease Inhibitor Cocktail III - EDTA-free (pH 8.0) for the HisTag-Ku70 protein. The suspension was sonicated seven times for 15 sec at 1 min intervals using output level 6 (Ultrasonic homogenizer: UH-50, SMT Company) and centrifuged at 12,000 × *g* for 20 min at 4 °C. The supernatant was mixed with 4 ml of 50% Glutathione Sepharose 4B (GE Healthcare) for GST-Htt protein or with 50% Ni-NTA Agarose (Qiagen) for HisTag-Ku70 protein, equilibrated with PBS containing 0.1% Tween 20, and rotated slowly for 3 hours at 4 °C. The GST-Htt suspension was applied to a Glutathione Sepharose 4B column at 4 °C, washed with 32 ml of PBS containing 0.1% Tween 20 (pH 8.0), and eluted four times with 4 ml of 10 mM glutathione in PBS containing 0.1% Tween 20 at 4 °C by gravity flow. The HisTag-Ku70 suspension was applied to a Ni-NTA Agarose column at 4 °C, washed with 32 ml of 20 mM imidazole in PBS containing 1% Triton X-100 (pH 8.0), and eluted four times with 4 ml of 10 mM glutathione in 250 mM imidazole in PBS containing 1% Triton X-100 (pH 8.0) at 4 °C by gravity flow. Each fraction was dialyzed and stirred with 2 L of PBS containing 0.01% Tween 20 for 12 hours at 4 °C twice. The purified Ku70 protein was labeled with fluorescent dye using a Protein Labeling Kit (488 nm and 633 nm) (Olympus).

### Single molecule fluorescence spectroscopy

Fluorescence intensity distribution analysis polarization (FIDA–PO) and fluorescence correlation spectroscopy (FCS) analyses were performed using an MF20 (Olympus Corporation), which detects the single-molecule fluorescence in 40 μl/well samples of 384-well glass-bottom plates using a confocal laser microscope at room temperature. The samples contained 5 nM fluorescent Ku70 protein in PBS containing 0.01% Tween 20 and 1% DMSO. The data of FIDA-PO and FCS were acquired from 10 times of experiments per well and the results from two wells were analyzed using the Bonferroni/Dunn test. In total, 18,500 chemicals from the Chemical Biology Screening Center of Tokyo Medical and Dental University were screened.

### *In silico* screening

All simulations were performed using Discovery Studio 2.5 (Dassault Systems BIOVIA Cooperation, San Diego) with a database of commercially available compounds, Chemical Available Purchase (CAP) 2006 (Dassault Systems BIOVIA Cooperation, San Diego). The structures of poly(amino acids) (fewer than ten residues) were generated using the command ‘Build and Edit Protein’ and were used for the binding simulation in Discovery Studio 3.0.

A crystal structure of the Ku heterodimer bound to DNA (PDB ID: 1JEY) was downloaded from the Research Collaboratory for Structural Bioinformatics (RCSB) Protein Data Bank (PDB), and the crystal structure of isolated Ku70 was imported from the complex (1JEY). The “prepare protein protocol” in Discovery Studio 3.0 was used to prepare the Ku70 structure for further processing. The protocol standardizes atoms names, applies the CHARMm (Chemistry at HARvard Macromolecular Mechanics) force field, and protonates amino acid residues except the inserts of missing loops. During preparation, default parameters were used, except ‘Build Loops:False’. The prepared Ku70 was used to define the binding site through the ‘Find Sites from Receptor Cavities’ option on the ‘Define and Edit Binding Site’ tool bar. Here, default parameters were used except ‘Site Opening: from 5.0 Å to 10.0 Å′. Chemicals were treated using the built-in ligand preparation wizard of Discovery Studio 2.5. Default parameters were used except ‘General Tautomers: False, General isomer: False, Fix Bad Valences: False’. In total, 3,010,121 chemicals were collected from CAP 2006 and examined using LibDock, a high-throughput algorithm for docking ligands into the binding site on the receptor^48^ to guide docking.

### Fly screening

All flies were raised on corn meal medium (9.2% corn meal, 3.85% yeast, 3.8% sucrose, 1.05% potassium tartrate, 0.09% calcium chloride, 7.6% glucose, 2.416% nipagin, and 1% agar) and maintained at 25 °C and 60% ± 10% humidity under a 12:12-h light-dark cycle unless otherwise noted.

UAS-Htt103Q and OK6-Gal4^49^ transgenic flies were crossed, and the F1 virgin female flies were subjected to lifespan screening. The chemicals or peptides for screening were dissolved in D.W. or ethanol at 5 mM and homogeneously mixed with 9 times the volume of corn meal medium to obtain a final concentration of 500 μM. For the control medium, only D.W. or ethanol was added. Twenty virgin female flies were maintained per vial and transferred to new vials with fresh medium every 2–3 days. The number of dead flies was quantified every 2–3 days.

### Administration of chemical compounds to R6/2 and mutant Htt-KI mice

Male R6/2 mice and their background mice (CBA/J) were maintained at 22 °C on a 12 h light/dark cycle (light on at 8:00 am and off at 8:00 pm) and had free access to water and standard chow pellets (CLEA Rodent Diet CE-2, CLEA Japan, Inc., Tokyo, Japan) prior to the start of the experiments. The mice received an intraperitoneal injection of a chemical compound dissolved in PBS at 50 μg/g body weight once a week from 3 weeks of age. Survival ratios were analyzed using the log-rank test. Mutant human Htt gene-KI mice^34^ (Hdh^*Q111*^ mice) were generous gift from Prof. Marcy MacDonald (Harvard Medical School). They were maintained and similarly and received an intraperitoneal injection of 7H dissolved in PBS at 50 μg/g body weight everyday for one week from 54 weeks of age.

### Western blotting analysis

The cerebral cortical tissues of 12-week-old mice were washed three times with ice-cold PBS and dissolved in lysis buffer containing 62.5 mM Tris-HCl, pH 6.8, 2% (w/v) SDS, 2.5% (v/v) 2-mercaptoethanol, 5% (v/v) glycerol, and 0.0025% (w/v) bromophenol blue. The protein concentration was quantified using the BCA method (Pierce BCA Protein Assay Kit; Thermo Scientific, IL, USA). These samples were separated by SDS-PAGE, transferred to an Immobilon-P polyvinylidene difluoride membrane (Millipore, MA, USA) through a semi-dry method, blocked by 5% milk in TBST (10 mM Tris/HCl, pH 8.0, 150 mM NaCl, and 0.05% Tween 20). Primary and secondary antibodies were diluted in TBST with 0.5% skim milk or Can Get Signal solution (Toyobo, Osaka, Japan) as follows: mouse anti-γH2AX, 1:1,000 (Ser139, #05-636, Millipore, MA, USA); rabbit anti-53BP1, 1:30,000 (NB100–304, Novus Biologicals, CO, USA); rabbit anti-Htt (CAG53b), 1:100,000 (a generous gift from Prof. Erich Wanker, Max Delbrück Center for Molecular Medicine, Berlin, Germany); anti-GAPDH, 1:1,000–10,000 (MAB374, Millipore; HRP-linked anti-rabbit IgG, 1:3,000 (NA934, GE Healthcare, Little Chalfont, United Kingdom); and HRP-linked anti-mouse IgG, 1:3,000 (NA931, GE Healthcare, Little Chalfont, United Kingdom). Primary and secondary antibodies were incubated overnight at 4 °C and for one hour at room temperature, respectively. ECL Prime Western Blotting Detection Reagent (RPN2232, GE Healthcare, Little Chalfont, United Kingdom) and luminescent image analyzer (ImageQuant LAS 500, GE Healthcare, Little Chalfont, United Kingdom) were used to detect proteins.

### Mouse motor function

Mice were maintained at 22 °C on a 12 h light/dark cycle (lights on at 8:00 am and off at 8:00 pm) and had free access to water and standard chow pellets (CLEA Rodent Diet CE-2, CLEA Japan, Inc.). Survival curves were analyzed using the log-rank test.

For the rotarod test, the mice were placed on a rotating rod (shaft diameter: 3.2 cm, lane width: 5.7 cm, fall height: 16.5 cm; Five Station Rotarod Standalone for Mouse, ENV-577M, MED Associates Inc., USA), and the speed of rotation was increased linearly from 0 to 35 rpm in 300 sec and maintained at 35 rpm for an additional 60 sec (for 4- to 12-week-old mice). The mice were examined in three trials performed on three consecutive days. The mean latency to fall off the rotarod in the three trials was recorded.

### Mouse immunohistochemistry

Immunohistochemical analysis was performed using mouse brains fixed in 4% paraformaldehyde for 12 to 16 hours. Mouse paraffin sections were deparaffinized in xylene followed by rehydration in ethanol diluted in serial dilutions and microwaved in 0.01 M citrate buffer, pH 6.0, at 120 °C for 15 min. The brain area was delimited using a DAKO pen (S2002, Agilent’s Dako, Glostrup, Denmark) and incubated overnight at 4 °C with primary antibodies. The next day, mouse brain sections were washed and incubated with secondary antibodies at room temperature for 1 hour. The antibodies and respective dilutions used included anti-γH2AX antibody, 1:200 (Ser139, #05-636, Millipore, MA, USA); rabbit anti-53BP1, 1:5000 (NB100-304, Novus Biologicals, CO, USA); anti-DARPP-32, 1:200 (Cell Signaling Technology, #2306, MA, USA); anti-Htt, 1:100 (EM48, #MAB5374, Millipore, MA, USA); anti-rabbit IgG Cy3, 1:1000 (711-165-152, Jackson Laboratory, Bar Harbor, ME, USA); anti-mouse IgG Alexa488, 1:1000 (A21202, Molecular Probes, OR, USA); anti-mouse IgG Cy3, 1:1000 (715-165-150, Jackson Laboratory, ME, USA); and anti-rabbit IgG Alexa488, 1:1000 (A21206, Molecular Probes, OR, USA).

### DNA-dependent protein kinase activity assay

The experiment was performed as described previously^24^. In brief, DNA-PK activity was measured using SignaTECT DNA-dependent Protein Kinase Assay System (Promega, #V7870, WI, USA) and ADP-Glo Kinase Assay (Promega, #V9101, WI, USA). Nuclear extracts of HEK293 cells were obtained using Nuclear Extract kit (Active Motif, #40010, CA, USA). Briefly, three peptides (7H, AngIII, LH-RH 4–10) were independently added into the reaction buffer mixture (DNA-PK activation buffer, DNA-PK 5xReaction buffer, DNA-PK biotinylated peptide substrate, BSA (66 μg/ml) and ATP (16.5 μM)), and after 10 minutes incubation at 30 °C, nuclear extracts were added. Kinase reaction was terminated by addition of ADP-Glo reagent. After ATP was removed, ADP remained was converted again to ATP by Detection Reagent, and the synthetized ATP was detected by luciferase reaction.

### Pull-down assay

Nuclear extracts of HeLa cell were obtained in accordance with our previous experimental protocol^24^. His-Ku70 or His-Tau (10 μg) were mixed with nuclear extracts and incubated for 12 hours at 4 °C, added with 50% Ni-NTA agarose (100 μL, Qiagen, Hilden, Germany), rotated for 3 hours at 4 °C, centrifuged at 500 *g* and washed with PBS (three times), and solubilized with sample buffer (62.5 mM Tris-HCl, pH 6.8, 2% (w/v) SDS, 2.5% (v/v) 2-mercaptoethanol, 5% (v/v) glycerol, and 0.0025% (w/v) bromophenol blue). The samples were subjected to Western blotting following the method described above. Primary and secondary antibodies were used as follows: mouse anti-His antibody (diluted at 1:3000, BioDynamics Laboratory Inc., #F008, Tokyo, Japan), anti-rabbit anti-ku80 (1:1000; H300; Santa Cruz Biotechnology, Inc. Texas, USA), anti-mouse IgG HRP-conjugated (1:3000, NA931, GE Healthcare, Little Chalfont, United Kingdom). Primary and secondary antibodies were incubated overnight at 4 °C and for one hour at room temperature, respectively.

### Common structure expectation

The six compounds, including Kemptide acetate salt (CID; 100074), Angiotensin III (CID; 71464372), Nordihydroguaiaretate (CID; 4534), Luteinizing hormone-releasing hormone fragment (CID; 4224599), 7H (hepta-histidine), and 7Q (hepta-glutamine), were multiply aligned using the flexible alignment method of Molecular Operating Environment (MOE) (2013.08; Chemical Computing Group Inc., Montreal, QC, Canada) with the default parameter setting. We employed the top scoring alignment among 100 candidates generated by the method to search for potential pharmacophores using the Pharmacophore Consensus application of MOE. In this study, the pharmacophore was defined as an atom that was common to at least five compounds in the alignment. Consequently, four pharmacophores were identified. One of the pharmacophores was a hydrogen bond acceptor (F1), and three of the pharmacophores were hydrogen bond donors (F2–F4).

### CD spectra

To investigate the secondary structures of the 3 peptides, 7H, AngIII, and LH-RH 4–10 fragment, their CD spectra were taken at 25 °C on a Jasco J-720 spectropolarimeter using an optical cuvette with a pathlength of 1.00 mm for measurements in the peptide region (200–250 nm). The concentrations of the peptides were 74–106 μM in PBS, which were estimated spectrophotometrically by applying the typical formula^50^. The concentrations of HFIP were set at 10 and 20% by adding appropriate amount of HFIP into the peptide solutions.

### Dynamic light scattering analysis

Dynamic light scattering (DLS) was performed on a Zetasizer μV instrument (Malvern Instruments Ltd.) to monitor the aggregation time course of huntingtin in the presence and absence of the drugs. Ten micromolar protein was incubated with 500 μM of each of the three drugs at 25 °C in PBS buffer for 6 days, and its DLS signal was recorded every several hours. The term “Z-average size” was applied as an index of aggregation (ISO-22412:2008). Each measurement was repeated four times, and the average of the four measurements was calculated.

### Culture of iPS cells

201B7 was derived from RIKEN BRC (https://ja.brc.riken.jp). CS92iHD-57n9 iPS cells of HD patients had been established in the Coriell Institute (https://catalog.coriell.org). CAG repeat number was 57.

### Karyotyping of iPS cells

Standard G-banding analysis of iPS cell lines (201B7 and CS92iHD-57n9) was performed to rule out the possibility of abnormal karyotypes that can occur during the generation of iPSCs.

### *In vitro* differentiation of iPS cells

Neural differentiation of iPS cells was performed as previously described^51^ with minor modifications. Briefly, the iPS cells were plated on a 10-cm dish and maintained for longer than 5 days with 3 μM SB431542, 3 μM CHIR99021 and 3 μM deosomorphine. Next, iPS cells were detached from a feeder layer and dissociated into single cells, which were cultured in 2 × B27 supplemented KBM medium (KHOJIN BIO, Saitama Japan) with 20 ng/ml bFGF, 10 ng/ml hLIF, 10 μM Y27632, 3 μM CHIR99021 and 2 μM SB431542 in a suspension culture condition in a 10-cm cell-repellent dish to form neurospheres. Neurospheres were passaged 2 times and differentiated into neural cells using an adhesion culture method (DMEM/F12 supplemented with B27 and Glutamax). Neurons were allowed to adhere to poly-L-ornithine- and poly-L-Lysine-coated coverslips for 14–21 days.

### Immunostaining of iPS and differentiated cells

Cells were fixed with PBS containing 4% paraformaldehyde for 15 min on ice and incubated with primary antibodies against the following proteins: SSEA1 (1:1000, Abcam, ab16285), Nanog (1:200, RCAB0004PF ReproCELL, Yokohama, Japan), βIII tubulin (1:1000, T8660 Sigma Chemical Co., MO, USA), αSMA (1:150, M085101 Dako, Glostrup, Denmark), SOX17 (1:500, ab84990 Abcam, Cambridge, UK), DARPP-32 (1:200, #2302S, Cell signaling, MA, USA), Cux1 (1:200, sc-13024, Santa Cruz Biotechnology, Inc., TX, USA) and TBR1 (1:200, ab31940, Abcam, Cambridge, UK). The cells were then washed with PBS and incubated with an Alexa Fluor 488-, Alexa Fluor 555-, or Alexa Fluor 647-conjugated secondary antibody (1:500, Invitrogen, CA, USA).

### Statistics

For lifespan assays of fly and mouse models, Log-rank test was used. For the other biological analyses, the data were considered to follow a normal distribution and are represented as the mean ± standard error. Student’s t-test was applied for two group comparisons (a chemical-treated group v.s. PBS group). For multiple group comparisons, Tukey’s HSD test or Dunnett’s comparison were applied. The significance level was set at 1% or 5%.

The power of statistical test was determined as the probability that the null hypothesis was correctly rejected. The power was estimated using sample size, significance level and effect size (ES). Effect size was calculated using Cohen’s d value^52^.

### Ethics

This study was performed in strict accordance with the recommendations in the Guide for the Care and Use of Laboratory Animals of the National Institutes of Health. It was approved by the Committees on Gene Recombination Experiments, Human Ethics, and Animal Experiments of the Tokyo Medical and Dental University (Numbers: 2010-215C14, 2014-5-4 and 0160328C, respectively).

## Additional Information

**How to cite this article**: Imamura, T. *et al*. Identification of hepta-histidine as a candidate drug for Huntington’s disease by *in silico-in vitro-in vivo*-integrated screens of chemical libraries. *Sci. Rep.* **6**, 33861; doi: 10.1038/srep33861 (2016).

## Supplementary Material

Supplementary Information
