# Supplementary material for: Identification of hepta-histidine as a candidate drug for Huntington’s disease by in silico-in vitro- in vivo-integrated screens of chemical libraries
Source: Sci Rep. 2016 Sep 22;6:33861. doi: 10.1038/srep33861 (PMC5032119; doi:10.1038/srep33861)
Supplement: Supplementary Information [file srep33861-s1.pdf]

Identification of hepta-histidine as a candidate drug for Huntington's disease by *in silico-in vitro-in vivo*-integrated screens of chemical libraries

Tomomi Imamura[1, $$], Kyota Fujita[1, $$], Kazuhiko Tagawa[1, $$], Teikichi Ikura[2 $$], Xigui Chen[1], Hidenori Homma[1], Takuya Tamura[1], Ying Mao[1], Juliana Bosso Taniguchi[1], Kazumi Motoki[1], Makoto Nakabayashi[2], Nobutoshi Ito[2], Kazunori Yamada[3], Kentaro Tomii[4], Hideyuki Okano[5], Julia Kaye[6], Steven Finkbeiner[6] and Hitoshi Okazawa[1, 7, #]

1: Department of Neuropathology, Medical Research Institute, Tokyo Medical and Dental University, 1-5-45 Yushima, Bunkyo-ku, Tokyo 113-8510, Japan

2: Department of Structural Biology, Medical Research Institute, Tokyo Medical and Dental University, 1-5-45 Yushima, Bunkyo-ku, Tokyo 113-8510, Japan

3: Graduate School of Information Sciences, Tohoku University, 6-3-09, Aramaki-Aza-Aoba, Aoba-ku, Sendai 980-8579, Japan

4: Cellular System Analysis Team, Computational Biology Research Center, National Institute of Advanced Industrial Science and Technology, 2-4-7, Aomi, Koto-ku, Tokyo 135-0064, Japan

5: Keio University School of Medicine, 35 Shinanomachi, Shinjuku-ku, Tokyo 112-0012, Japan.

6: Taube/Koret Center for Neurodegenerative Disease, Gladstone Institute of Neurological Disease, and the Departments of Neurology and Physiology, University of California San Francisco, San Francisco CA 94158, USA

7: Center for Brain Integration Research, Tokyo Medical and Dental University, 1-5-45 Yushima, Bunkyo-ku, Tokyo 113-8510, Japan

$$: TI, KF, KT, and TI contributed equally to this manuscript.

#: Corresponding author

Supplementary Figure 1

Single molecule fluorescence spectroscopy

(A) Basic principle of single molecule fluorescence spectroscopy (MF20). Ku70 protein was labeled with fluorescent dye using a Protein Labeling Kit (488 nm and 633 nm) (Olympus) before analysis with MF20. The change in the fluorescence parameters of a single molecule of Ku70 (the first molecule chemically labeled to have fluorescence) caused by an interaction with another molecule such mutant Htt (the second non-florescent molecule) in a very small space (1 x 10^{-15} L) was detected within 20-60 sec/well.

This figure is modified from the original image made by Olympus Co. Ltd., under the permission.

(B) Principles underlying two parameters, diffusion time and fluorescence polarization. An interaction with the second molecule resulted in an increase in both parameters.

(C) Preliminary experiments to establish the appropriate conditions for detecting the increase in the two parameters caused by the interaction between Ku70 (fluorescent) and mutant Htt (non-fluorescent). FCS: fluorescence correlation spectroscopy reflecting diffusion time. FIDA-PO: fluorescence intensity distribution analysis-polarization reflecting fluorescence polarization. Mean +/- S.D. are shown in graph.

(D) A representative set of raw data from the screening of LMW chemicals.

(E) Pull down assay to test whether bacterially expressed Ku70-HisTag keeps its physiological function to interact with endogenous Ku80. Ku70-HisTag was mixed with HEK293 nuclear extract and Ni-Sepharose was used to pull down Ku70-HisTag. The output shows interaction of Ku70-HisTag but not Tau-HisTag with endogenous Ku80.

Supplementary Figure 2

Raw data of the 2nd screening with MF20 for the chemicals selected from the 1st screening with MF20

The results of 2nd screen of the chemical from 1st screen with MF20.are shown. Value of polarization in FIDA-PO in the binding state between Ku70 and mutant

Htt is shown with yellow bar as a positive control, and that of negative control (Ku70 only) is shown in red bar. When addition of a chemical statistically decreased the value of FIDA-PO from that of Ku70+Htt110Q to a value between yellow bar and red bar levels at least at one concentration, the chemical was selected as a candidate to dissociate the interaction between Ku70 and mutant Htt. Data acquisition was repeated 10 times per well and the results from 2 wells were collectively used for statistical analysis. Mean +/- S.E. are shown in graph. Single saterisk (*) indicates $p<0.05$ and double asterisks (**) indicates $p<0.01$ in Student's t-test followed by Bonferroni's correction.

Supplementary Figure 3

Raw data of the 2nd screening with MF20 for the chemicals selected from the 1st screening with Discovery Studio

The results of 2nd screen of the chemical from 1st screen with Discovery Studio are shown. The 2nd screen exactly followed the method of the 1st screen with MF20.

Supplementary Figure 4

Raw data of the 3rd screening with a *Drosophila* model

The 3rd screen was performed with a Drosophila Gal4-UAS model overexpressing human mutant Htt Exon1-103Q in motor neurons by OK6 driver[49]. UAS-Htt103Q and OK6-Gal4 transgenic flies were crossed, and the F1 virgin female flies were fed with chemicals or peptides that were dissolved in D.W. or ethanol at 5 mM and filuted with 9 times volume of corn meal medium to a final concentration at 500 μM. Only D.W. or ethanol was added to the controls. Twenty virgin female flies were maintained per vial and transferred to new vials with fresh medium every 2–3 days. The number of dead flies was quantified every 2–3 days.

In Sup Fig 4, only negative chemicals in 3rd screen are shown. Positive chemicals passed through the 3rd screening are shown in Figure 4A.

Supplementary Figure 5

Effect of candidate chemicals on DNA damage and mutant Htt aggregation in vivo

(A) Staining for the DNA double-strand break marker, γH2AX, in striatal neurons (NeuN-positive) and striatal medium spiny neurons (DARPP32-positive) of R6/2 mice fed #4028 (Angiotensin III), hepta-histidine and L5387 (LH-RH 4-10 peptide fragment).

(B) Recovery of 53BP1 in the same neurons.

(C) Inclusion body formation was tested using immunohistochemistry with anti-Htt antibody (EM48). The three chemicals did not decrease the neuronal aggregation of mutant Htt.

(D) Western blotting analyses of cortical tissues using DNA damage markers revealed that oral administration of #4028 (Angiotensin III) and hepta-histidine but not L5387 (LH-RH 4-10 peptide fragment) reduced DNA damage.

(E) The left panels of western blots with anti-Htt antibody or anti-ubiquitin antibody revealed that the chemicals did not largely affect the aggregation or poly-ubiquitination processes. The right graphs show the quantities of HMW aggregates (indicated with box) reactive for anti-Htt or anti-Ub antibody corrected with the signal intensities of GAPDH. No significant change was observed by statistical test with Student's t-test or Tukey's HSD test.

Supplementary Figure 6

Subtype characterization of neurons differentiated from human iPS cells

Subtypes of neurons differentiated from iPS cells were characterized with neuron-subtype-specific markers, DARPP32, Cux1 and TBR1. The values from ten visual fields are summarized as mean +/- S.E.

Supplementary Figure 7

Effect of the final candidate chemicals on DNA-PK activity

Each chemical was added into the reaction at final concentration of 50 or 100μM in which DNA-PK phosphorylates the substrate (see the method for the details). The extent of phosphorylation was not suppressed by any of the three peptides. Instead, phosphorylation of the substrate by DNA-PK was enhanced by 7H.

Supplementary Table 1

List of chemicals that were selected from the first screening with MF20 and forwarded to the second screening with MF20

Supplementary Table 2

List of chemicals that were selected from the first screening with Discovery Studio and forwarded to the second screening with MF20

Supplementary Table 3

List of chemicals that were selected from the second screening with MF20 and forwarded to the third screening with the *Drosophila* HD model

Supplementary Figure 1

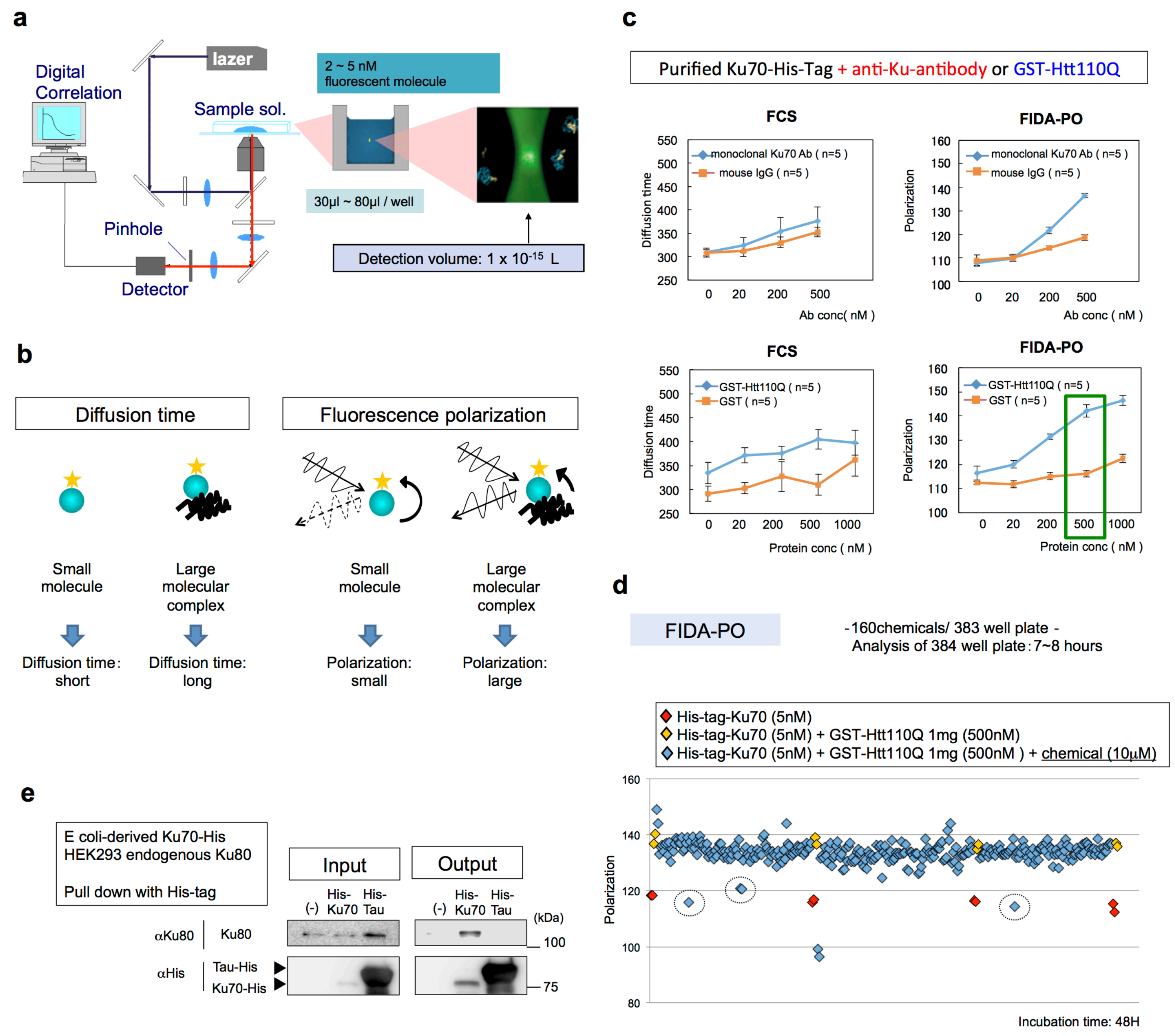

Supplementary Figure 2

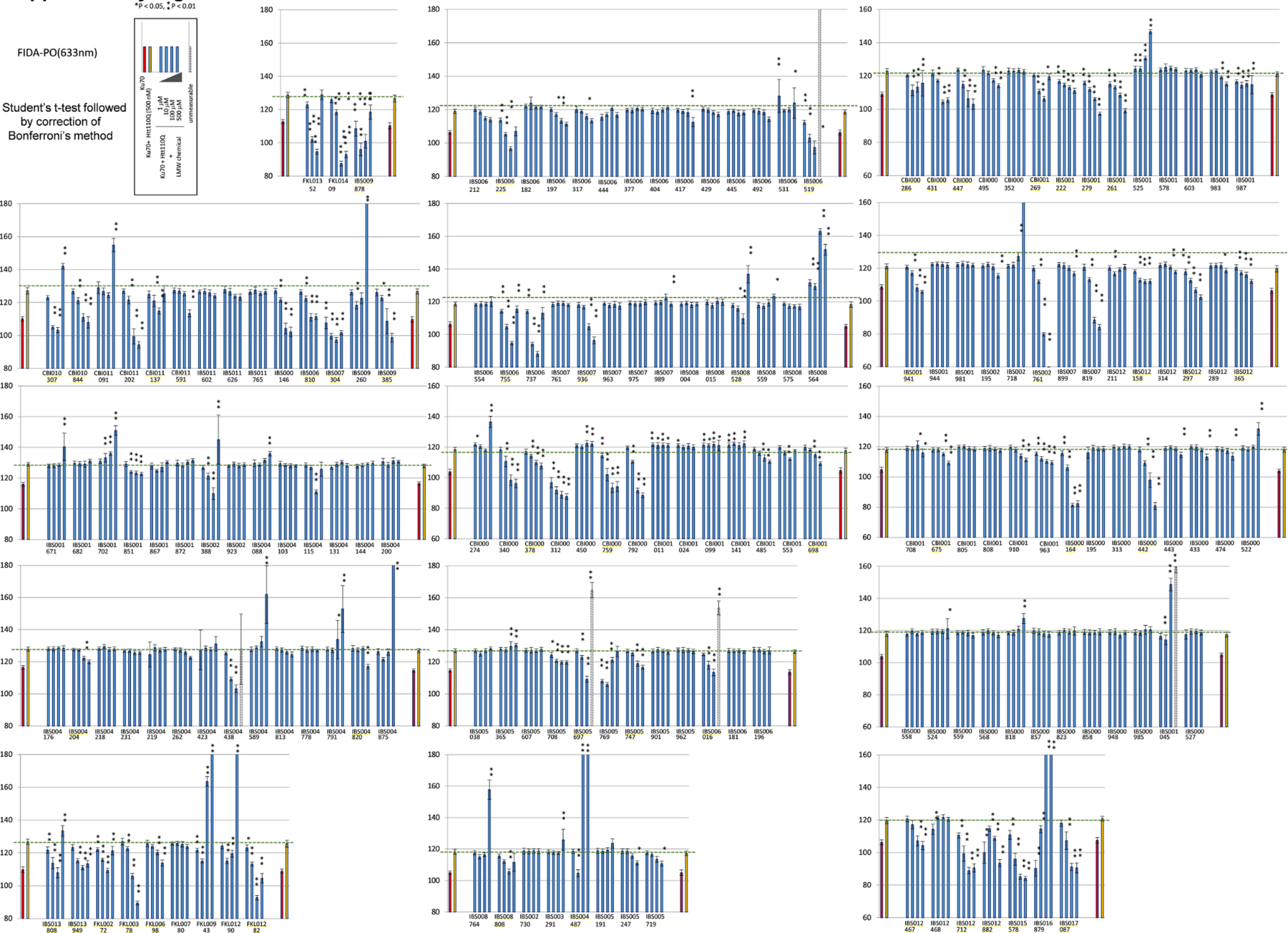

Supplementary Figure 3

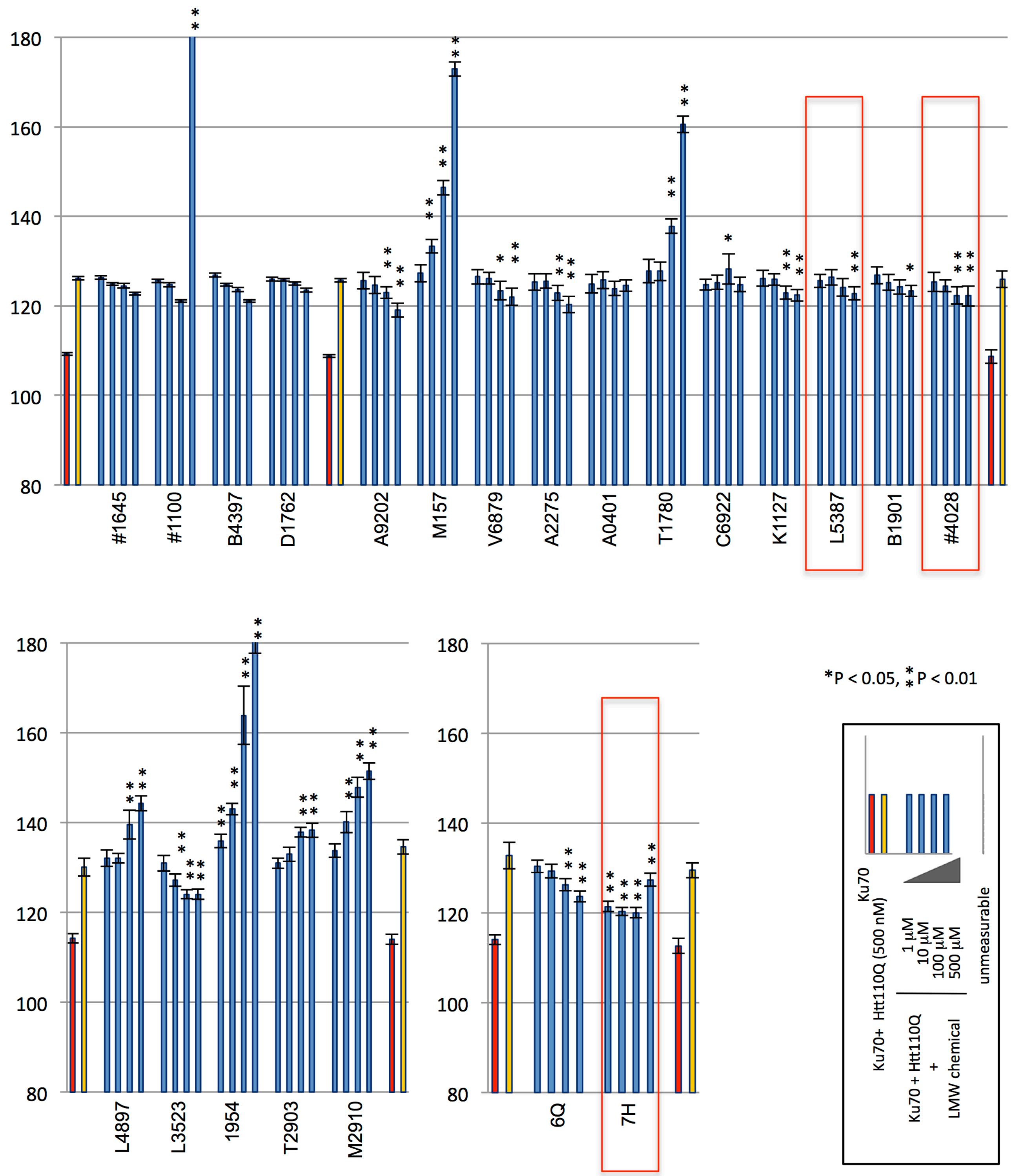

Supplementary Figure 4

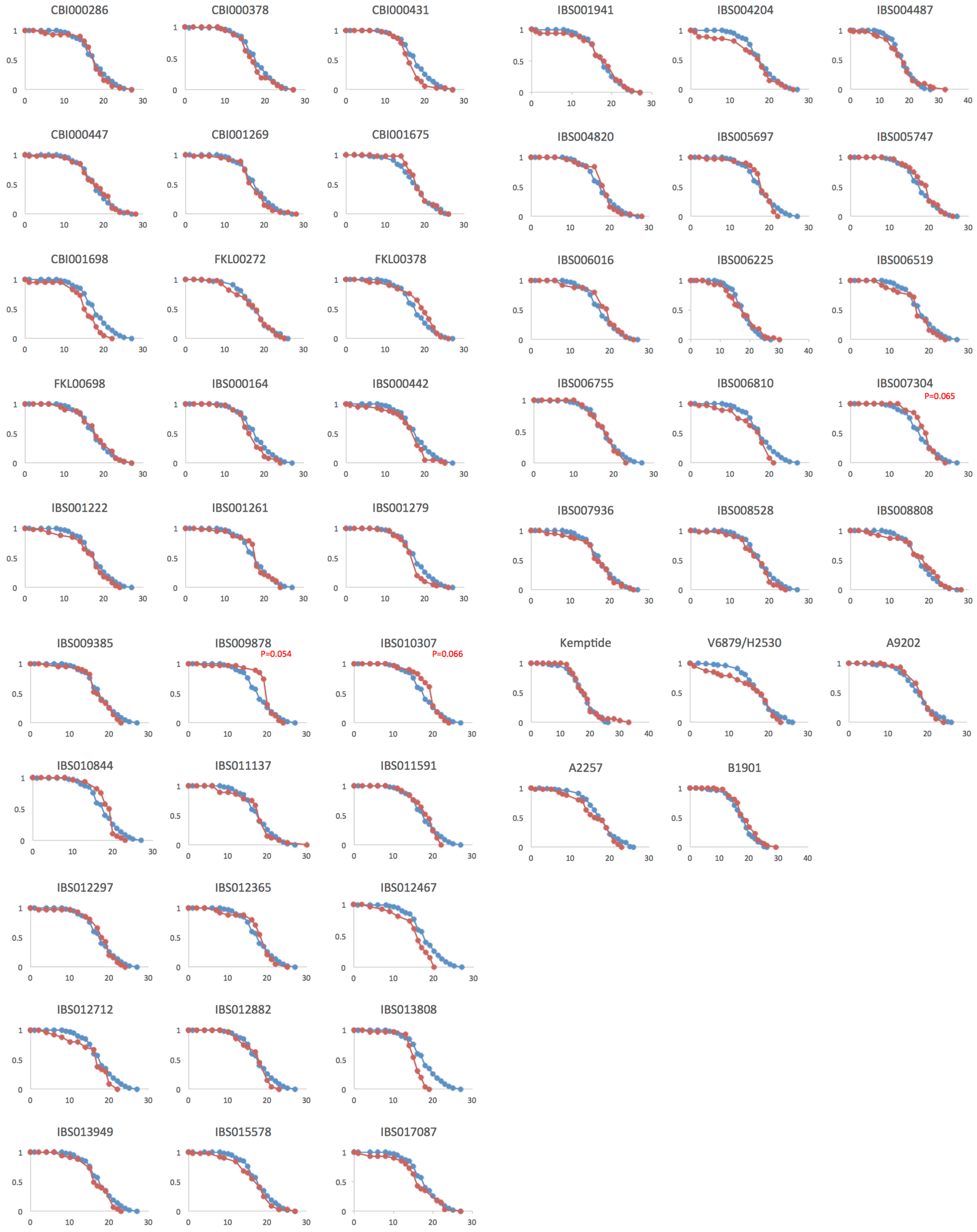

Supplementary Figure 5

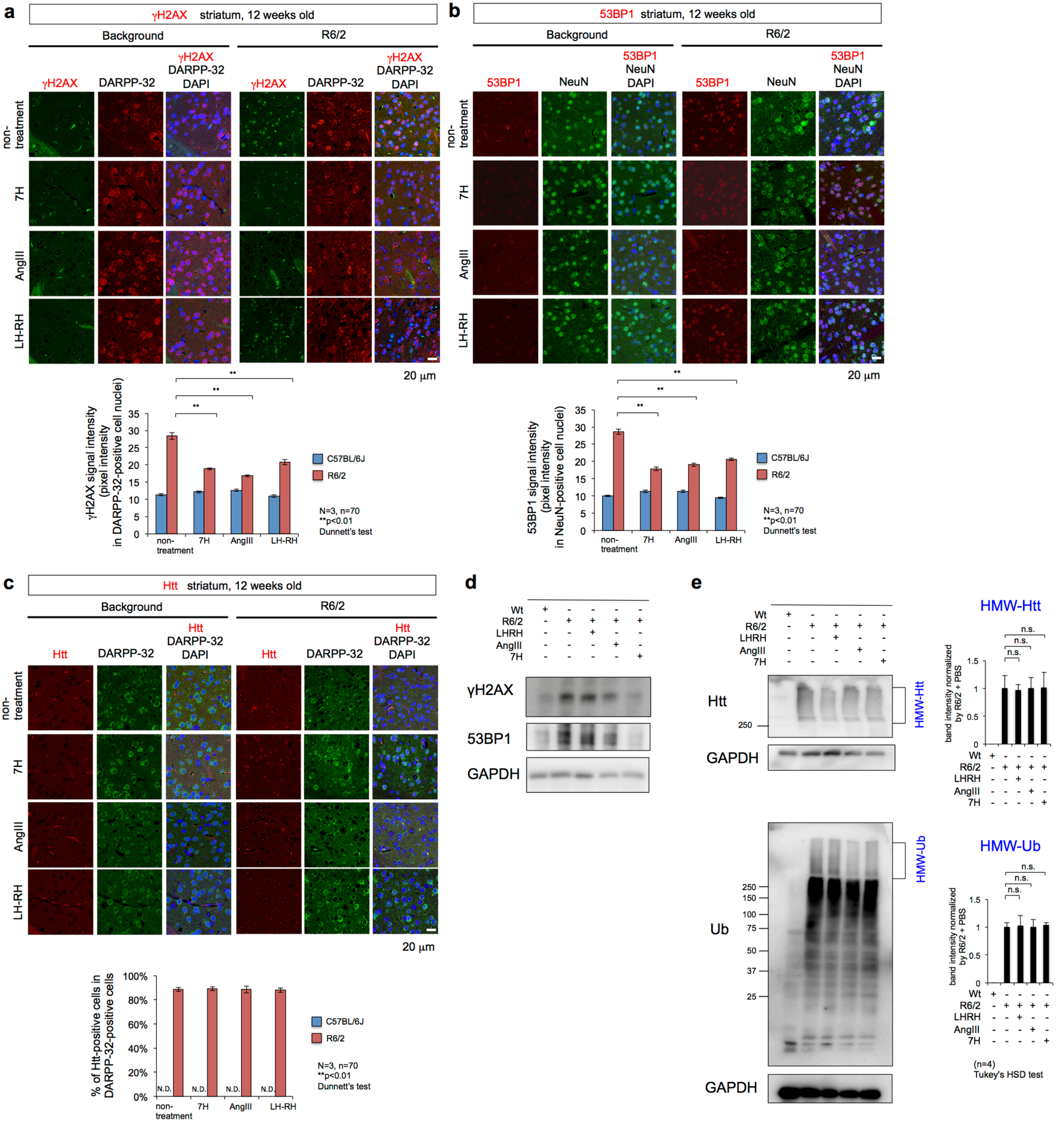

Supplementary Figure 6

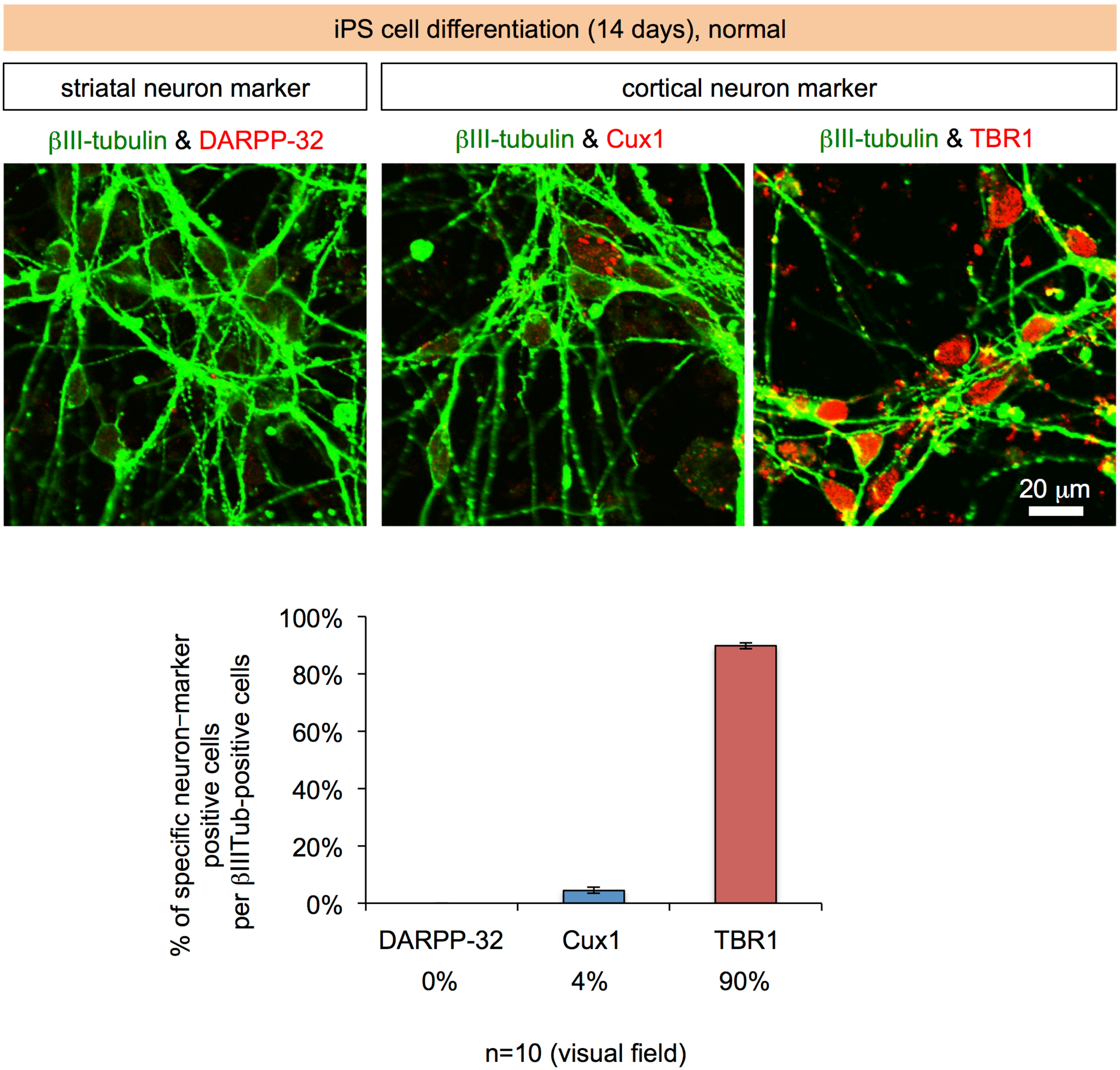

Supplementary Figure 7

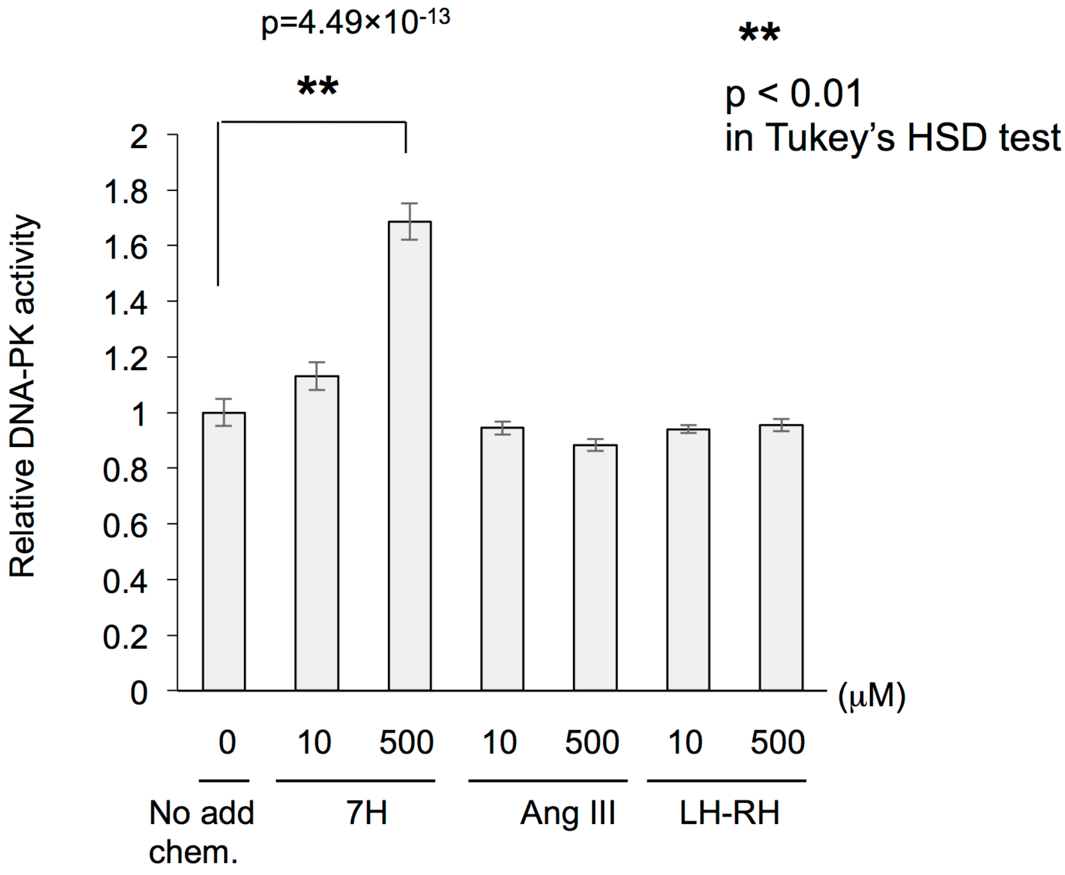

Supplementary Table 1

Product Number	CAS No.	Molecular Weight	Name	IUPAC
CBI000146	-	280.238		6, 7- di(furan- 2- yl) pteridin- 4- ol
CBI000274	-	260.272		(E) - 2- ((Z) - (2- oxoindolin- 3- ylidene) hydrazono) thiazolidin- 4- one
CBI000286	-	335.193		1,1'',4'-trinitro-1H,1'H,1''H-4,3':5',4''-terpyrazole
CBI000312	-	270.181		[N-(1,3-dihydro-2H-benzimidazol-2-ylidene-kappaN~1~)ethanimidamidato-kappaN'](dipropyl)boron
CBI000340	5351-91-7	185.27		(E) - 2- (thiophen- 2- ylmethylene) hydrazinecarbothioamide
CBI000352	-	285.252		1,8-dichloro-3,4,5,6,9,10-hexamethyltricyclo[6.2.0.0~3,6~]deca-4,9-diene
CBI000378	53266-96-9	212.269		ethyl 3- methyl- 5, 6- dihydroimidazo [2, 1- b] thiazole- 2- carboxylate
CBI000431	-	312.517		9, 9a- dihydro- 4aH- [1, 3] dithiolo [4, 5- b] indeno [2, 1- e] [1, 4] dithiine- 2- thione
CBI000447	-	296.473		5, 7- dihydrospiro [[1, 3] dithiolo [4, 5- b] [1, 4] dithiepine- 6, 2'- [1, 3] dioxolane] - 2- thione
CBI000450	-	203.237		8-(propan-2-ylidene)-3a,4,7,7a-tetrahydro-1H-4,7-methanoisoindole-1,3(2H)-dione
CBI000495	-	249.335		9- (piperidin- 1- ylmethyl) - 1H- purine- 6(9H) - thione
CBI000527	37132-72-2	431.353		2,2,4,4,6-pentakis(aziridin-1-yl)-6-(morpholin-4-yl)-1,3,5,2lambda~5~,4lambda~5~,6lambda~5~-triazatriphosphinine
CBI000759	-	198.606		(1E, 2Z) - N- hydroxy- 2- (hydroxyimino) - 2- phenylacetimidoyl chloride
CBI000792	3702-88-3	181.106		6- nitrobenzo [c] [1, 2, 5] oxadiazole 1- oxide
CBI001011	-	214.225		N,N-dimethyl-2,3-dihydro-1,3,2-benzothiazaphosphol-2-amine 2-oxide
CBI001024	-	448.578		7, 9- dichloro- 4- (4- chlorophenyl) - 2, 2- bis(trifluoromethyl) - 2H- pyrido [1, 2- a] [1, 3, 5] triazine
CBI001099	-	296.344		2-(pyridin-2-ylmethyl)-2H-naphtho[1,8-cd][1,2]thiazole 1,1-dioxide
CBI001141	-	189.259		4- amino- 3- methyl- 2- thioxo- 2, 3- dihydrothiazole- 5- carboxamide
CBI001269	-	308.406		10, 11- dimethylthieno [3, 2- e] bis [1, 2, 4] triazolo [4, 3- a:4', 3'- c] pyrimidine- 3, 7(2H, 6H) - dithione
CBI001485	-	238.241		2- phenylquinoxaline 1, 4- dioxide
CBI001553	-	279.229		4-hydroxy-8-nitrothieno[2,3-b:4,5-b']dipyridine-2,7(1H,6H)-dione
CBI001675	-	234.301		[1, 2, 4] triazolo [4, 3- a] quinoxaline- 1, 4- dithiol
CBI001698	-	283.37		3- (morpholinomethyl) - 5- (thiophen- 2- yl) - 1, 3, 4- oxadiazole- 2(3H) - thione
CBI001708	-	420.385		5-bromo-N-[2-(tricyclo[3.3.1.1~3,7~]dec-1-yloxy)ethyl]thiophene-2-sulfonamide
CBI001805	4580-63-6	171.26		1- thia- 4- azaspiro [4.5] decan- 3- one
CBI001808	-	197.218		7- amino- 3- methyl- [1, 2, 4] triazino [3, 4- b] [1, 3, 4] thiadiazin- 4(8H) - one
CBI001910	-	229.366		5- methyl- 3- (piperidin- 1- ylmethyl) - 1, 3, 4- thiadiazole- 2(3H) - thione
CBI001963	-	131.09		2- (hydroxyimino) malonamide
FKL00272	41372-20-7	303.783	Apomorphine hydrochlorid	(6aR)-6-methyl-5,6,6a,7-tetrahydro-4H-dibenzo[de,g]quinoline-10,11-diol;hydrochloride
FKL00378	62-31-7	189.639	Dopamine hydrochloride	4-(2-aminoethyl)benzene-1,2-diol;hydrochloride
FKL00698	49745-95-1	337.841	Dobutamine hydrochloride	4-[2-[4-(4-hydroxyphenyl)butan-2-ylamino]ethyl]benzene-1,2-diol;hydrochloride
FKL00780	3198-07-0	233.692	Ethylnorepinephrine hydrochloride	[1-(3,4-dihydroxyphenyl)-1-hydroxybutan-2-yl]azanium;chloride
FKL00943	16423-68-0	879.856	Erythrosine sodium	disodium;2',4',5',7'-tetraiodo-3-oxospiro[2-benzofuran-1,9'-xanthene]-3',6'-diolate
FKL01282	500-38-9	302.365	Nordihydroguaiaretate	4-[(2S,3S)-4-(3,4-dihydroxyphenyl)-2,3-dimethylbutyl]benzene-1,2-diol
FKL01290	480-16-0	302.236	Morin	2-(2,4-dihydroxyphenyl)-3,5,7-trihydroxychromen-4-one
FKL01352	491-67-8	270.237	Baicalein	5,6,7-trihydroxy-2-phenylchromen-4-one
FKL01409	6954-48-9	237.050	Bonaphton	6-bromonaphthalene-1,2-dione
IBS000164	-	350.411		3- (1- hydroxy- 3, 3- dimethyl- 3, 4, 10, 11- tetrahydro- 2H- dibenzo [b, e] [1, 4] diazepin- 11- yl) benzene- 1, 2- diol
IBS000195	-	458.348		7- bromo- 4- (3- morpholinopropanoyl) - 5- phenyl- 4, 5- dihydro- 1H- benzo [e] [1, 4] diazepin- 2(3H) - one
IBS000313	-	297.305		(E) - ethyl 4- (benzo [d] [1, 3] dioxol- 5- ylmethyleneamino) benzoate
IBS000433	-	283.386		4- (2- (2, 6- dimethoxyphenoxy) ethyl) thiomorpholine

Product Number	CAS No.	Molecular Weight	Name	IUPAC
IBS000442	-	293.408		1- (morpholinomethyl) quinoxaline- 2, 3(1H, 4H) - dithione
IBS000443	-	338.402		dimethyl 2, 2'- (quinoxaline- 2, 3- diylbis(sulfanediyl)) diacetate
IBS000474	-	224.323		2, 2, 5, 5- tetramethyl- 4- (thiophen- 2- yl) - 2, 5- dihydro- 1H- imidazol- 1- ol
IBS000522	-	281.372		1, 6- dimethyl- 2- (2- oxo- 2- p- tolylethyl) - 3, 4- dihydropyrrolo [1, 2- a] pyrazin- 2- ium
IBS000524	-	252.309		2- (5- (thiophene- 2- carbonyl) thiophen- 2- yl) acetic acid
IBS000558	-	215.274		2- methyl- 6- phenylimidazo [2, 1- b] [1, 3, 4] thiadiazole
IBS000559	-	280.278		(Z) - 7- hydroxy- 2- (phenylimino) - 2H- chromene- 3- carboxamide
IBS000568	-	293.317		3- methyl- 2- (quinolin- 2- yl) - 2, 3- dihydrobenzo [b] [1, 4] dioxin- 2- ol
IBS000818	-	293.706		ethyl 4- (4- chlorophenylcarbamoyl) - 1H- imidazole- 5- carboxylate
IBS000823	-	326.143		methyl 4- (3- amino- 5- bromobenzofuran- 2- yl) - 4- oxobutanoate
IBS000857	-	283.322		3- (indoline- 1- carbonyl) bicyclo [2.2.1] hept- 5- ene- 2- carboxylic acid
IBS000858	-	252.372		1- (9- acetoxybicyclo [3.3.1] nonan- 2- yl) pyrrolidinium
IBS000948	-	220.2		2- (4- (4- fluorophenyl) - 1H- imidazol- 1- yl) acetic acid
IBS000985	-	327.335		methyl 2- (4- hydroxy- 1- (4- methoxyphenyl) - 3- methyl- 1H- pyrazolo [3, 4- b] pyridin- 6- yl) acetate
IBS001045	-	301.383		(Z) - 4- thioxo- 5- ((5- p- tolylfuran- 2- yl) methylene) thiazolidin- 2- one
IBS001222	-	214.175		(7E, 9Z) - 7, 9- bis(hydroxyimino) - 1, 4- dioxaspiro [4.5] decan- 8- one
IBS001261	-	191.187		2- methyl- 1H- indole- 4, 5- dione dioxime
IBS001279	-	276.245		(3E, 5Z) - 3, 5- bis(hydroxyimino) - 4- oxocyclohexyl benzoate
IBS001525	-	358.415		ethyl 5- methyl- 3- (2- (thiazol- 2- ylamino) acetamido) - 1H- indole- 2- carboxylate
IBS001578	-	376.432		2- (2- amino- 5- (2- phenylthiazol- 4- yl) pyrimidin- 4- yl) - 5- methoxyphenol
IBS001603	-	187.322		1- (3, 3- dimethyl- 2- oxobutyl) tetrahydro- 1H- thiophenium
IBS001671	-	525.558		N-[3-(carbamoylamino)-3-oxo-2-(triphenyl-lambda~5~-phosphanylidene)propanethioyl]benzamide
IBS001682	-	343.446		6- chloro- 5- (2, 3- dibromopropyl) - 2- methylpyrimidin- 4- amine
IBS001702	-	286.322		3- (4- methoxyphenoxy) - 1- (4- methoxyphenyl) propan- 1- one
IBS001851	-	377.373		diisopropyl 2- amino- 1- (cyclohexylcarbamoyloxyimino) - 2- oxoethylphosphonate
IBS001867	-	342.284		(E) - diethyl 2- amino- 1- (2- methylbenzoyloxyimino) - 2- oxoethylphosphonate
IBS001872	-	343.117		1- acetyl- 5- iodo- 1H- indol- 3- yl acetate
IBS001941	-	312.019		(2E, 6Z) - 2, 6- bis(3, 3- dichloroallylidene) cyclohexanone
IBS001944	-	435.982		3, 8- bis(iodomethyl) - 2, 7- dioxaspiro [4.4] nonane- 1, 6- dione
IBS001981	-	364.335		(S) - ethyl 3, 3, 3- trifluoro- 2- (4- fluorophenethylamino) - 2- propionamidopropanoate
IBS001983	-	251.235		sodium (E) - 2- (benzylideneamino) ethyl sulfate
IBS001987	51210-89-0	253.253		2- (hydroxyimino) - 1, 3- diphenylpropane- 1, 3- dione
IBS002195	-	331.146		N- (benzo [c] [1, 2, 5] oxadiazol- 4- yl) - 2, 2, 3, 3, 4, 4, 4- heptafluorobutanamide
IBS002388	-	298.403		1- allyl- 3- (3- phenoxybenzyl) thiourea
IBS002718	-	346.419		4- (3- (5- methylfuran- 2- yl) benzofuran- 2- yl) - 2- phenylbutan- 2- ol
IBS002730	-	332.268		diethyl 5, 10- dioxo- 5, 10- dihydrodiimidazo [1, 5- a:1', 5'- d] pyrazine- 1, 6- dicarboxylate
IBS002761	70402-14-1	195.217		6- amino- 1H- phenalen- 1- one
IBS002923	-	393.372		2- ((2- acetamidophenyl) (phenyl) phosphoryl) phenyl acetate
IBS003291	-	422.548		2, 2'- (1, 3, 4- thiadiazole- 2, 5- diyl) bis(sulfanediyl) bis(1- (3, 5- dimethyl- 1H- pyrazol- 1- yl) ethanone)
IBS004088	-	365.426		2- (3- (3, 5- dimethyl- 1H- pyrazol- 1- yl) - 2- hydroxypropoxy) - N- phenylbenzamide
IBS004103	-	281.261		3, 3'- (3- carboxyphenylazanediyl) dipropanoic acid
IBS004115	-	310.271		2- (2, 3, 5- trifluoro- 6- (pyrrolidin- 1- yl) pyridin- 4- yloxy) phenol
IBS004131	-	327.22		4- (5- bromo- 2- (piperidin- 1- yl) pyrimidin- 4- yl) morpholine
IBS004144	-	324.495		4,7,8-trichloro-1,3-difluoro[1,4]benzodioxino[2,3-c]pyridine
IBS004176	98299-40-2	331.568		cyclohexyl(phenyl) (3- (piperidin- 1- yl) propyl) silanol

Product Number	CAS No.	Molecular Weight	Name	IUPAC
IBS004200	-	458.511		2- (benzo [d] oxazol- 2- ylthio) - N- (4- (N- (3, 4- dimethylisoxazol- 5- yl) sulfamoyl) phenyl) acetamide
IBS004204	-	263.355		3- (2- oxo- 2- ((1S, 2S, 4S) - 1, 7, 7- trimethylbicyclo [2.2.1] heptan- 2- yloxy) ethyl) - 1H- imidazol- 3- ium
IBS004218	-	160.601		1- (5- chloro- 1- methyl- 1H- imidazol- 2- yl) ethanol
IBS004219	-	344.385		3- ((benzo [d] thiazol- 2- ylamino) methylene) - 1, 5- dioxaspiro [5.5] undecane- 2, 4- dione
IBS004231	-	279.401		5- cyclohexyl- 1- (furan- 2- ylmethyl) - 1, 3, 5- triazinane- 2- thione
IBS004262	-	303.379		5- oxo- N- phenethyl- 3, 5, 6, 7- tetrahydro- 2H- imidazo [2, 1- b] [1, 3] thiazine- 7- carboxamide
IBS004423	-	391.43		(Z) - 5- ((E) - 3- phenylallylidene) - 2- thioxo- 3- (3- (trifluoromethyl) phenyl) thiazolidin- 4- one
IBS004438	-	527.576		4-{10-methyl-6-[(4-methylphenyl)amino]-8-(pyridin-2-yl)-8,11-dihydropyrazolo[3',4':4,5]pyrimido[1,2-a]quinoxalin-11-yl}benzene-1,2-diol
IBS004487	-	492.547		N- ((tetrahydrofuran- 2- yl) methyl) - 2- (3- ((2, 4, 6- trioxo- 1- (thiophen- 2- ylmethyl) tetrahydropyrimidin- 5(6H) - ylidene) methyl) - 1H- indol- 1- yl) acetamide
IBS004589	-	468.549		(Z) - 3- (benzylthio) - 5- imino- 6- (2, 3, 4- trimethoxybenzylidene) - 5H- [1, 2, 4] thiadiazolo [4, 5- a] pyrimidin- 7(6H) - one
IBS004778	-	389.414		(4aS, 9bR) - 2, 8- dimethyl- N- (3- (trifluoromethyl) phenyl) - 2, 3, 4, 4a- tetrahydro- 1H- pyrido [4, 3- b] indole- 5(9bH) - carboxamide
IBS004791	-	507.507		4- (4- (4- methoxyphenylsulfonyl) piperazin- 1- yl) - 4- oxo- N- (5- (trifluoromethyl) - 1, 3, 4- thiadiazol- 2- yl) butanamide
IBS004813	-	300.354		(4- (1H- indol- 3- yl) pyridin- 1(4H) - yl) (phenyl) methanone
IBS004820	-	342.369		2-[(3-phenyl-4,5-dihydro-1,2-oxazol-5-yl)methyl]-1,2-benzothiazol-3(2H)-one 1,1-dioxide
IBS004875	-	511.658		2-[(8-methyl-7-oxo-5,6,6b,7,8,10a-hexahydronaphtho[2',1':4,5]thieno[2,3-d]pyrimidin-9-yl)sulfanyl]-N,N-diphenylacetamide
IBS005038	-	277.216		N-(3-oxido-3,4-dihydro-2H-1,5,3-benzodioxaphosphepin-3-yl)pyrimidin-2-amine
IBS005191	-	394.465		3'- phenyl- 1', 2', 3', 4', 4a', 6'- hexahydrospiro [indene- 2, 5'- pyrazino [1, 2- a] quinoline] - 1, 3- dione
IBS005247	-	472.536		3-(3,5-dimethoxyphenyl)-5-propyl-3H-pyrrolo[2',3':4,5]pyrimido[1,6-a]benzimidazol-1-yl butanoate
IBS005365	-	405.465		N- (3- (4- fluorophenyl) - 3- (furan- 2- yl) propyl) - 3- (2- methyl- 1H- benzo [d] imidazol- 1- yl) propanamide
IBS005607	-	302.324		dimethyl 2- (4- (ethoxycarbonyl) piperazin- 1- yl) succinate
IBS005697	-	422.457		4- ethyl- N- (2- (4- methoxyphenylcarbamoyl) benzofuran- 3- yl) - 1, 2, 3- thiadiazole- 5- carboxamide
IBS005708	14337-43-0	151.548		(Z) - ethyl 2- chloro- 2- (hydroxyimino) acetate
IBS005719	857041-65-7	225.354		2- (4- methylpiperazin- 1- yl) - 2- (thiophen- 2- yl) ethanamine
IBS005747	-	294.333		(E) - N- (3- (1H- imidazol- 1- yl) propyl) - 2- (2- aminothiazol- 4- yl) - 2- (hydroxyimino) acetamide
IBS005769	-	314.339		ethyl 3- (1- (1H- benzo [d] imidazol- 2- yl) - 5- hydroxy- 3- methyl- 1H- pyrazol- 4- yl) propanoate
IBS005901	-	204.185		3- (2- methyl- 2H- tetrazol- 5- yl) benzoic acid
IBS005962		251.260		2- (4- fluorobenzylamino) - 1- (2- methyl- 2H- tetrazol- 5- yl) ethanol
IBS006016		401.502		ethyl 2- (2- (thiophen- 2- yl) acetamido) - 4- p- tolylthiazol- 5- ylcarbamate
IBS006181	-	343.301		N- (1- isopropyl- 1H- pyrazol- 5- yl) - 2- (4- (trifluoromethoxy) phenoxy) acetamide
IBS006182	-	382.413		N- (1- methyl- 2- (2- (tetrahydrofuran- 2- carboxamido) ethyl) - 1H- benzo [d] imidazol- 5- yl) furan- 2- carboxamide
IBS006196	-	278.307		N1- (2- morpholinoethyl) - N2- (pyridin- 3- yl) oxalamide
IBS006197	-	259.325		4-{[3-(1H-imidazol-1-yl)propyl]amino}tetrahydrothiophene-3-ol 1,1-dioxide
IBS006212	-	287.314		(Z) - N'- hydroxy- 1- (3- methoxyphenyl) - 4, 6- dimethyl- 2- oxo- 1, 2- dihydropyridine- 3- carboximidamide
IBS006225	-	256.217		5- (4- (1H- tetrazol- 1- yl) phenyl) furan- 2- carboxylic acid
IBS006317	-	262.306		2- (3- butoxy- 2- hydroxypropyl) malonohydrazide

Product Number	CAS No.	Molecular Weight	Name	IUPAC
IBS006377	-	239.233		(E) - 5- (phenyldiazenyl) benzo [c] [1, 2, 5] oxadiazol- 4- amine
IBS006404	-	216.238		(Z) - 1, 2- bis((E) - 1- (hydroxyimino) - 2- methylpropan- 2- yl) diazene oxide
IBS006417	-	318.281		(5S)-5,6-dihydroxy-2-(3-nitrophenyl)hexahydro-1H-4,7-methanoisoindole-1,3(2H)-dione
IBS006429	89607-12-5	205.997		4- bromo- 1- methyl- 3- nitro- 1H- pyrazole
IBS006444	33050-32-7	170.557		6- chloro- [1, 2, 4] triazolo [4, 3- b] pyridazin- 3(2H) - one
IBS006445	-	394.768		11- (4- chlorophenyl) - 2, 4- dinitro- 5H- dibenzo [b, e] [1, 4] diazepine
IBS006492	-	327.446		2- isopentyl- 2- (2- methylthiazol- 4- yl) pentanedihydrazide
IBS006519	-	513.523		(E) - methyl 5- ((S, Z) - 4- benzamido- 3- (2- (3, 5- dinitrophenyl) hydrazono) dihydrothiophen- 2(3H) - ylidene) pentanoate
IBS006531	-	350.287		N- ((2- (2, 4- dinitrophenyl) hydrazono) methyl) - 2- (2- oxopyrrolidin- 1- yl) acetamide
IBS006554	-	293.345		(E)-1-(5-nitrothiophen-2-yl)-N-(1,3,5-triazatricyclo[3.3.1.1~3,7~]dec-7-yl)methanimine
IBS006737	-	284.267		(E) - 2- (2- (benzylamino) - 1- nitrovinyl) cyclohexa- 2, 5- diene- 1, 4- dione
IBS006755	-	238.24		2- (2- (isopropylamino) - 1- nitrovinyl) benzene- 1, 4- diol
IBS006810	-	301.342		2- (1H- benzo [d] imidazol- 2- yl) - N- (4- fluorophenyl) hydrazinecarbothioamide
IBS007304	-	352.495		ethyl 5'- (3- allylthioureido) - 2, 3'- bithiophene- 4'- carboxylate
IBS007761	-	282.183		N- (1, 1, 1, 3, 3, 3- hexafluoro- 2- (2- methoxyethylamino) propan- 2- yl) acetamide
IBS007819	-	436.419		3- hydroxy- 4- (4- nitrophenyl) - 2- phenyl- 4, 10- dihydroindeno [2, 1- e] pyrazolo [3, 4- b] pyridin- 5(2H) - one
IBS007899	-	299.209		2, 2, 3, 3, 4, 4, 4- heptafluoro- 1- thiomorpholinobutan- 1- one
IBS007936	-	470.611		4- amino- N- (4- (dimethylamino) phenyl) - 2- (2- (4- (dimethylamino) phenylamino) - 2- oxoethylthio) thiazole- 5- carboxamide
IBS007963	-	324.294		(S) - 4, 4, 4- trifluoro- 3- hydroxy- 1- (2- hydroxy- 5- methylphenyl) - 3- phenylbutan- 1- one
IBS007975	-	391.41		morpholino(3- (naphthalen- 2- yl) - 5- (trifluoromethyl) - 1H- pyrazol- 1- yl) methanethione
IBS007989	-	180.121		6- nitro- 1H- benzo [d] [1, 2, 3] triazol- 1- ol
IBS008004	-	303.323		N- cyclohexyl- N- methyl- 2- (5- methyl- 3- (trifluoromethyl) - 1H- pyrazol- 1- yl) acetamide
IBS008015	-	298.293		(E) - 4- (2- benzoylhydrazono) - 4, 5, 6, 7- tetrahydrobenzofuran- 2- carboxylic acid
IBS008528	-	299.348		2- (4- hydroxyphenylamino) - 5- ((1- methyl- 1H- pyrrol- 2- yl) methylene) thiazol- 4(5H) - one
IBS008559	-	321.438		2- (3, 4- dimethoxybenzyl) - 5, 5, 7- trimethyl- 5, 6- dihydro- 2H- 1, 2, 4- triazepine- 3(4H) - thione
IBS008564	-	276.293		1- (1H- benzo [d] imidazol- 2- yl) - 3- phenyl- 1H- pyrazol- 5- ol
IBS008575	-	397.537		2-{[5-(4-aminophenyl)-4-methyl-4H-1,2,4-triazol-3-yl]sulfanyl}-N-(tricyclo[3.3.1.1~3,7~]dec-1-yl)acetamide
IBS008764	-	347.417		N- (3- (1H- imidazol- 1- yl) propyl) - 5, 6- dimethyl- 7- (pyridin- 2- yl) - 7H- pyrrolo [2, 3- d] pyrimidin- 4- amine
IBS008808	-	359.443		(E) - N- (2- (5, 6- dihydroimidazo [2, 1- b] thiazol- 3- yl) ethyl) - 3- (3, 4- dimethoxyphenyl) acrylamide
IBS009260	-	375.336		6-{(E)-2-[4-(diethylamino)-3-nitrophenyl]ethenyl}-5-nitropyrimidine-2,4-diol
IBS009385	-	475.968		2-({[(5Z)-5-(2-chlorobenzylidene)-2,4-dioxo-1,3-thiazolidin-3-yl]acetyl}amino)-4,5,6,7-tetrahydro-1-benzothiophene-3-carboxamide
IBS009878	-	396.459		(2Z)-5,5-dimethyl-2-({[(4-methylphenyl)sulfonyl]oxy}imino)-5,6-dihydropyrrolo[2,1-a]isoquinolin-3(2H)-one
IBS010307	-	306.267		ethyl 5-[2-oxo-2-(2,3,4-trihydroxyphenyl)ethyl]furan-2-carboxylate
IBS010844	-	389.203		N'-[(E)-(5-bromo-2-hydroxyphenyl)methylidene]-3-(5-methylfuran-2-yl)-1H-pyrazole-5-carbohydrazide
IBS011091	-	416.472		2-[(4Z)-2,5-dioxo-4-{[1-(propan-2-yl)-1H-indol-3-yl]methylidene}imidazolidin-1-yl]-N-(4-methylphenyl)acetamide

Product Number	CAS No.	Molecular Weight	Name	IUPAC
IBS011137	-	543.615		ethyl 1-[(3-{2-[(2E)-2-(3-nitrobenzylidene)hydrazinyl]-1,3-thiazol-4-yl}phenyl)sulfonyl]piperidine-4-carboxylate
IBS011202	-	464.624		(4Z)-2-methoxy-7,7-dimethyl-4-(4-oxo-2-thioxo-1,3-thiazolidin-5-ylidene)-10-thioxo-7,10-dihydro[1,2]dithiolo[3,4-c]pyrrolo[3,2,1-ij]quinolin-5(4H)-one
IBS011591	-	196.208		2-(pyridin-3-yl)-2H-benzotriazole
IBS011602	-	345.356		4-amino-N-(2-{[(1,3-dimethyl-2-oxo-2,3-dihydro-1H-benzimidazol-5-yl)methyl]amino}ethyl)-1,2,5-oxadiazole-3-carboxamide
IBS011626	-	260.315		5-(thiophen-2-yl)-3-(1H-1,2,4-triazol-5-ylamino)cyclohex-2-en-1-one
IBS011765	-	336.388		6-(4-methoxybenzyl)-3-[(1-phenylethyl)amino]-1,2,4-triazin-5(4H)-one
IBS012158	29917-12-2	129.114		3- hydroxyiminopentane- 2, 4- dione
IBS012211	41601-44-9	162.192		4,6-dimethyl-1H-pyrazolo[3,4-b]pyridin-3-amine
IBS012289	1011408-19-7	182.206		2- methylsulfanyl- [1, 2, 4] triazolo [1, 5- a] [1, 3, 5] triazin- 7- amine
IBS012297	-	184.113		(5Z,6Z)-N,N'-dihydroxy[1,2,5]oxadiazolo[3,4-b]pyrazine-5,6(4H,7H)-diimine
IBS012314	-	188.188		[amino- [(3, 5- dioxo- 2H- 1, 2, 4- triazin- 6- yl) sulfanyl] methylidene] azanium
IBS012365	-	200.142		(2E) - N- (3, 4- difluorophenyl) - 2- hydroxyiminoacetamide
IBS012467	41153-83-7	215.004		6-bromo-2,1,3-benzoxadiazole 1-oxide
IBS012468	35330-57-5	215.066		4-bromotetrahydrothiophene-3-ol 1,1-dioxide
IBS012712	-	241.249		(6E)-N-hydroxy-5-methyl-7-phenyl[1,2,4]triazolo[1,5-a]pyrimidin-6(7H)-imine
IBS012882	-	254.241		(2E)-N-(dibenzo[b,d]furan-2-yl)-2-(hydroxyimino)ethanamide
IBS013808	23821-62-7	301.383		[2-phenyl-4-(thiophen-2-yl)-1,3-thiazol-5-yl]acetic acid
IBS013949	-	307.386		(1R,6S) - N - {[(4 - fluorophenyl)carbamothioyl]amino}bicyclo[4.1.0]heptane - 7 - carboxamide
IBS015578	-	362.596		4,4,4-trichloro-3-(1H-imidazol-1-yl)-1-(4-nitrophenyl)butan-1-one
IBS016879	-	402.466		5-(furan-2-ylmethylidene)-1,3-bis(2-methylphenyl)-2-thioxodihydropyrimidine-4,6(1H,5H)-dione
IBS017087	-	408.407		N'-[(Z)-(4-hydroxy-3-methoxy-5-nitrophenyl)methylidene]-2,3,4,9-tetrahydro-1H-carbazole-6-carbohydrazide

Supplementary Table 2

Library	DS Score	Product Number	CAS No.	Molecular Weight	Name	IUPAC
Chemical Library (CAP06)	214	SIGMA-L4897	86073-88-3	1212.314	Luteinizing Hormone-Releasing Hormone salmon (GnRH) (LH-RH)	N-[1-[[1-[[1-[[1-[[2-[[1-[[1-[2-[(2-amino-2-oxoethyl)carbamoyl]pyrrolidin-1-yl]-4-methyl-1-oxopentan-2-yl]amino]-3-(1H-indol-3-yl)-1-oxopropan-2-yl]amino]-2-oxoethyl]amino]-3-(4-hydroxyphenyl)-1-oxopropan-2-yl]amino]-3-hydroxy-1-oxopropan-2-yl]amino]-3-(1H-indol-3-yl)-1-oxopropan-2-yl]amino]-3-(1H-imidazol-5-yl)-1-oxopropan-2-yl]-5-oxopyrrolidine-2-carboxamide
Chemical Library (CAP06)	197	SIGMA-L3523	80224-16-4	887.040	[Trp4]-Kemptide (Leu-Arg-Arg-Trp-Ser-Leu-Gly)	2-[[2-[[2-[[2-[[2-[[2-[(2-amino-4-methylpentanoyl)amino]-5-(diaminomethylideneamino)pentanoyl]amino]-5-(diaminomethylideneamino)pentanoyl]amino]-3-(1H-indol-3-yl)propanoyl]amino]-3-hydroxypropanoyl]amino]-4-methylpentanoyl]amino]acetic acid
Chemical Library (CAP06)	195	TOCRIS-1954	115150-59-9	951.190	Antagonist G (Arg-DTrp-NMe-Phe-DTrp-Leu-Met-NH2) ([Arg6,D-Trp7,9,N-MePhe8]-Substance P(6-11))	(2S)-2-[[(2R)-2-[[(2S)-2-[[(2R)-2-[[(2S)-2-amino-5-(diaminomethylideneamino)pentanoyl]amino]-3-(1H-indol-3-yl)propanoyl]-methylamino]-3-phenylpropanoyl]amino]-3-(1H-indol-3-yl)propanoyl]amino]-N-[(2S)-1-amino-4-methylsulfanyl-1-oxobutan-2-yl]-4-methylpentanamide
Chemical Library (CAP06)	193	SIGMA-T2903	65418-88-4	882.0189	Trp-His-Trp-Leu-Gln-Leu (α1-Mating factor fragment 1-6)	2-[[5-amino-2-[[2-[[2-[[2-[[2-amino-3-(1H-indol-3-yl)propanoyl]amino]-3-(1H-imidazol-5-yl)propanoyl]amino]-3-(1H-indol-3-yl)propanoyl]amino]-4-methylpentanoyl]amino]-5-oxopentanoyl]amino]-4-methylpentanoic acid
Chemical Library (CAP06)	192	SIGMA-M2910	87616-84-0	873.014	[D-Trp7, Ala8, D-Phe10]-alpha-Melanocyte Stimulating Hormone Amide Fragment 6-11 (GHRP-6)	(2S)-6-amino-2-[[(2R)-2-[[(2S)-2-[[(2S)-2-[[(2R)-2-[[(2S)-2-amino-3-(1H-imidazol-5-yl)propanoyl]amino]-3-(1H-indol-3-yl)propanoyl]amino]propanoyl]amino]-3-(1H-indol-3-yl)propanoyl]amino]-3-phenylpropanoyl]amino]hexanamide
Chemical Library (CAP06)	191	SIGMA-V6879	50-57-7	1056.218	[Lys8]-Vasopressin	N-[6-amino-1-[(2-amino-2-oxoethyl)amino]-1-oxohexan-2-yl]-1-[19-amino-7-(2-amino-2-oxoethyl)-10-(3-amino-3-oxopropyl)-13-benzyl-16-[(4-hydroxyphenyl)methyl]-6,9,12,15,18-pentaoxo-1,2-dithia-5,8,11,14,17-pentazacycloicosane-4-carbonyl]pyrrolidine-2-carboxamide
Chemical Library (CAP06)	190	SIGMA-D1762	102783-42-6	966.404	P1,P5-Di(guanosine-5') pentaphosphate ammonium salt	azanium;bis[[[5-(2-amino-6-oxo-3H-purin-9-yl)-3,4-dihydroxyoxolan-2-yl]methoxy-hydroxyphosphoryl]oxy-hydroxyphosphoryl] hydrogen phosphate
Chemical Library (CAP06)	190	SIGMA-M157	135306-85-3	1081.224	MEN-10,376 (Tyr(5)-Trp(6,8,9)-Lys(10)-neurokinin A(4-10))	3-amino-4-[[1-[[1-[[1-[[1-[[1-[(1,6-diamino-1-oxohexan-2-yl)amino]-3-(1H-indol-3-yl)-1-oxopropan-2-yl]amino]-3-(1H-indol-3-yl)-1-oxopropan-2-yl]amino]-3-methyl-1-oxobutan-2-yl]amino]-3-(1H-indol-3-yl)-1-oxopropan-2-yl]amino]-3-(4-hydroxyphenyl)-1-oxopropan-2-yl]amino]-4-oxobutanoic acid
Chemical Library (CAP06)	190	BACHEM #N-1100	50410-01-0	1025.202	His-Pro-Phe-His-Leu-D-Leu-Val-Tyr	2-[[2-[[2-[[2-[[2-[[2-[[1-[2-amino-3-(1H-imidazol-5-yl)propanoyl]pyrrolidine-2-carbonyl]amino]-3-phenylpropanoyl]amino]-3-(1H-imidazol-5-yl)propanoyl]amino]-4-methylpentanoyl]amino]-4-methylpentanoyl]amino]-3-methylbutanoyl]amino]-3-(4-hydroxyphenyl)propanoic acid
Chemical Library (CAP06)	188	SIGMA-K1127	65189-71-1	771.908	Kemptide Acetate salt (for PKA) (Leu-Arg-Arg-Ala-Ser-Leu-Gly)	2-[[(2S)-2-[[(2S)-2-[[(2S)-2-[[(2S)-2-[[(2S)-2-[[(2S)-2-amino-4-methylpentanoyl]amino]-5-(diaminomethylideneamino)pentanoyl]amino]-5-(diaminomethylideneamino)pentanoyl]amino]propanoyl]amino]-3-hydroxypropanoyl]amino]-4-methylpentanoyl]amino]acetic acid

Screening	DS Score	Product Number	CAS No.	Molecular Weight	Name	IUPAC
Chemical Library (CAP06)	187	SIGMA-A9202	51833-78-4	899.005	Angiotensin Fragment 1-7 acetate salt hydrate (Asp-Arg-Val-Tyr-Ile-His-Pro)	1-[2-[[2-[[2-[[2-[[2-[(2-amino-3-carboxypropanoyl)amino]-5-(diaminomethylideneamino)pentanoyl]amino]-3-methylbutanoyl]amino]-3-(4-hydroxyphenyl)propanoyl]amino]-3-methylpentanoyl]amino]-3-(1H-imidazol-5-yl)propanoyl]pyrrolidine-2-carboxylic acid
Chemical Library (CAP06)	185	SIGMA-A2275	34273-10-4	912.047	[Sar1, Val5, Ala8]-Angiotensin II acetate salt hydrate (Saralasinum) (Sar-Arg-Val-Tyr-Val-His-Pro-Ala)	2-[[1-[2-[[2-[[2-[[2-[[5-(diaminomethylideneamino)-2-[[2-(methylamino)acetyl]amino]pentanoyl]amino]-3-methylbutanoyl]amino]-3-(4-hydroxyphenyl)propanoyl]amino]-3-methylbutanoyl]amino]-3-(1H-imidazol-5-yl)propanoyl]pyrrolidine-2-carbonyl]amino]propanoic acid
Chemical Library (CAP06)	184	SIGMA-A0401	4037-01-8	962.09	Adrenocorticotropic Hormone Fragment 4-10 human, rat	4-[(2-amino-4-methylsulfanylbutanoyl)amino]-5-[[1-[[1-[[1-[[1-(carboxymethylamino)-3-(1H-indol-3-yl)-1-oxopropan-2-yl]amino]-5-(diaminomethylideneamino)-1-oxopentan-2-yl]amino]-1-oxo-3-phenylpropan-2-yl]amino]-3-(1H-imidazol-5-yl)-1-oxopropan-2-yl]amino]-5-oxopentanoic acid
Chemical Library (CAP06)	182	SIGMA-B1901	16875-11-9	904.023	Bradykinin Fragment 2-9 (Pro-Pro-Gly-Phe-Ser-Pro-Phe-Arg)	5-(diaminomethylideneamino)-2-[[2-[[1-[3-hydroxy-2-[[3-phenyl-2-[[2-[[1-(pyrrolidine-2-carbonyl)pyrrolidine-2-carbonyl]amino]acetyl]amino]propanoyl]amino]propanoyl]pyrrolidine-2-carbonyl]amino]-3-phenylpropanoyl]amino]pentanoic acid
Chemical Library (CAP06)	181	SIGMA-T1780	6934-38-9	996.391	Tyr-Tyr-Tyr-Tyr-Tyr-Tyr (Hexa-L-tyrosine)	-[[2-[[2-[[2-[[2-[[2-amino-3-(4-hydroxyphenyl)propanoyl]amino]-3-(4-hydroxyphenyl)propanoyl]amino]-3-(4-hydroxyphenyl)propanoyl]amino]-3-(4-hydroxyphenyl)propanoyl]amino]-3-(4-hydroxyphenyl)propanoyl]amino]-3-(4-hydroxyphenyl)propanoic acid
Chemical Library (CAP06)	180	Peptide Inst. #4028	100900-06-9	931.091	Angiotensin III, Human (Arg-Val-Tyr-Ile-His-Pro-Phe)	2-[[1-[2-[[2-[[2-[[2-[[2-amino-5-(diaminomethylideneamino)pentanoyl]amino]-3-methylbutanoyl]amino]-3-(4-hydroxyphenyl)propanoyl]amino]-3-methylpentanoyl]amino]-3-(1H-imidazol-5-yl)propanoyl]pyrrolidine-2-carbonyl]amino]-3-phenylpropanoic acid
Chemical Library (CAP06)	179	BACHEM #H-1645	63555-63-5	823.940	Ala-Ser-His-Leu-Gly-Leu-Ala-Arg (complement C3a, 70-77)	(2S)-2-[[(2S)-2-[[(2S)-2-[[2-[[(2S)-2-[[(2S)-2-[[(2S)-2-[[(2S)-2-aminopropanoyl]amino]-3-hydroxypropanoyl]amino]-3-(4H-imidazol-4-yl)propanoyl]amino]-4-methylpentanoyl]amino]acetyl]amino]-4-methylpentanoyl]amino]propanoyl]amino]-5-(diaminomethylideneamino)pentanoic acid
Chemical Library (CAP06)	178	SIGMA-L5387	51776-33-1	747.842	Luteinizing Hormone-Releasing Hormone Fragment 4-10 (LH-RH Fragment 4-10, Ser-Tyr-Gly-Leu-Arg-Pro-Gly-NH2)	1-[2-[[2-[[2-[[2-[(2-amino-3-hydroxypropanoyl)amino]-3-(4-hydroxyphenyl)propanoyl]amino]acetyl]amino]-4-methylpentanoyl]amino]-5-(diaminomethylideneamino)pentanoyl]-N-(2-amino-2-oxoethyl)pyrrolidine-2-carboxamide
Chemical Library (CAP06)	176	SIGMA-C6922	113584-01-3	758.908	Cyclohexylacetyl-Phe-Arg-Ser-Val-Gln amide	2-[[2-[[2-[[2-[[2-[(2-cyclohexylacetyl)amino]-3-phenylpropanoyl]amino]-5-(diaminomethylideneamino)pentanoyl]amino]-3-hydroxypropanoyl]amino]-3-methylbutanoyl]amino]pentanediamide
Chemical Library (CAP06)	175	SIGMA-B4397	15958-92-6	904.023	Bradykinin Fragment 1-8 (des-Arg(9)-bradykinin) (Arg-Pro-Pro-Gly-Phe-Ser-Pro-Phe)	2-[[1-[2-[[2-[[2-[[1-[1-[2-amino-5-(diaminomethylideneamino)pentanoyl]pyrrolidine-2-carbonyl]pyrrolidine-2-carbonyl]amino]acetyl]amino]-3-phenylpropanoyl]amino]-3-hydroxypropanoyl]pyrrolidine-2-carbonyl]amino]-3-phenylpropanoic acid

Screening	DS Score	Product Number	CAS No.	Molecular Weight	Name	IUPAC
Poly A.A.	192			786.791	Gln-Gln-Gln-Gln-Gln-Gln (6Q)	
Poly A.A.	205			977.990	His-His-His-His-His-His-His (7H)	

Availability of chemicals selected from in silico screen

DS score	Product name	Campany Catalog No.
214.180	Luteinizing Hormone-Releasing Hormone salmon	SIGMA-L4897
197.061	[Trp4]-Kemptide Leu-Arg-Arg-Trp-Ser-Leu-Gly	SIGMA-L3523
195.273	Antagonist G Arg-DTrp-NMe-Phe-DTrp-Leu-Met-NH2	TOCRIS-1954
193.336	Trp-His-Trp-Leu-Gln-Leu	SIGMA-T2903
192.246	Tyr-D-Trp-Ala-Trp-D-Phe amide	Not available
192.209	[D-Trp7, Ala8, D-Phe10]-alpha-Melanocyte Stimulating Hormone Amide Fragment 6-11	SIGMA-M2910
191.283	[Lys8]-Vasopressin	SIGMA-V6879
190.201	P1,P5-Di(guanosine-5') pentaphosphate ammonium salt	SIGMA-D1762
189.897	MEN-10,376	SIGMA-M157
189.893	His-Pro-Phe-His-Leu-D-Leu-Val-Tyr	BACHEM #N-1100
188.458	Kemptide Acetate salt	SIGMA-K1127
186.601	Angiotensin Fragment 1-7	SIGMA-A9202
184.681	[Sar1, Val5, Ala8]-Angiotensin II	SIGMA-A2275
184.064	[des-Pro3, Ala2,6]-Bradykinin	Not available
183.757	Adrenocorticotropic Hormone Fragment 4-10 human, rat	SIGMA-A0401
181.967	Bradykinin Fragment 2-9	SIGMA-B1901
181.822	N-CBZ-L-Lysyl-L-lysyl-L-arginine 7-amido-4-methylcoumarin, triacetate salt	Not available
181.194	Tyr-Tyr-Tyr-Tyr-Tyr-Tyr	SIGMA-T1780
179.933	Angiotensin III	Peptide Inst. #4028v
179.439	Ala-Ser-His-Leu-Gly-Leu-Ala-Arg	BACHEM #H-1645
178.029	Luteinizing Hormone-Releasing Hormone Fragment 4-10	SIGMA-L5387
177.133	4-Nitrophenyl 2-O-(2',3',4',6'-tetra-O-4-methoxybenzyl-α-D-glucopyranosyl)-4,6-benzylidene-α-D-glucopyranoside	Not available
176.966	1. beta-Lipotropin Fragment 39-45	Not available
175.610	Cyclohexylacetyl-Phe-Arg-Ser-Val-Gln amide	SIGMA-C6922
175.408	Bradykinin Fragment 1-8	SIGMA-B4397

Supplementary Table 3

1st screening	Product Number	CAS No.	Molecular Weight	Name	IUPAC
MF20 / CAP06	CBI000286	-	335.193		1,1'',4'-trinitro-1H,1'H,1''H-4,3':5',4''-terpyrazole
MF20 / CAP06	CBI000378	53266-96-9	212.269		ethyl 3- methyl- 5, 6- dihydroimidazo [2, 1- b] thiazole- 2- carboxylate
MF20 / CAP06	CBI000431	-	312.517		9, 9a- dihydro- 4aH- [1, 3] dithiolo [4, 5- b] indeno [2, 1- e] [1, 4] dithiine- 2- thione
MF20 / CAP06	CBI000447	-	296.473		5, 7- dihydrospiro [[1, 3] dithiolo [4, 5- b] [1, 4] dithiepine- 6, 2'- [1, 3] dioxolane] - 2- thione
MF20 / CAP06	CBI000759	-	198.606		(1E, 2Z) - N- hydroxy- 2- (hydroxyimino) - 2- phenylacetimidoyl chloride
MF20 / CAP06	CBI001269	-	308.406		10, 11- dimethylthieno [3, 2- e] bis [1, 2, 4] triazolo [4, 3- a:4', 3'- c] pyrimidine- 3, 7(2H, 6H) - dithione
MF20 / CAP06	CBI001675	-	234.301		[1, 2, 4] triazolo [4, 3- a] quinoxaline- 1, 4- dithiol
MF20 / CAP06	CBI001698	-	283.37		3- (morpholinomethyl) - 5- (thiophen- 2- yl) - 1, 3, 4- oxadiazole- 2(3H) - thione
MF20 / CAP06	FKL00272	41372-20-7	303.783	Apomorphine hydrochlorid	(6aR)-6-methyl-5,6,6a,7-tetrahydro-4H-dibenzo[de,g]quinoline-10,11-diol;hydrochloride
MF20 / CAP06	FKL00378	62-31-7	189.639	Dopamine hydrochloride	4-(2-aminoethyl)benzene-1,2-diol;hydrochloride
MF20 / CAP06	FKL00698	49745-95-1	337.841	Dobutamine hydrochloride	4-[2-[4-(4-hydroxyphenyl)butan-2-ylamino]ethyl]benzene-1,2-diol;hydrochloride
MF20 / CAP06	FKL01282	500-38-9	302.365	Nordihydroguaiaretate	4-[(2S,3S)-4-(3,4-dihydroxyphenyl)-2,3-dimethylbutyl]benzene-1,2-diol
MF20 / CAP06	IBS000164	-	350.411		3- (1- hydroxy- 3, 3- dimethyl- 3, 4, 10, 11- tetrahydro- 2H- dibenzo [b, e] [1, 4] diazepin- 11- yl) benzene- 1, 2- diol
MF20 / CAP06	IBS000442	-	293.408		1- (morpholinomethyl) quinoxaline- 2, 3(1H, 4H) - dithione
MF20 / CAP06	IBS001222	-	214.175		(7E, 9Z) - 7, 9- bis(hydroxyimino) - 1, 4- dioxaspiro [4.5] decan- 8- one
MF20 / CAP06	IBS001261	-	191.187		2- methyl- 1H- indole- 4, 5- dione dioxime
MF20 / CAP06	IBS001279	-	276.245		(3E, 5Z) - 3, 5- bis(hydroxyimino) - 4- oxocyclohexyl benzoate
MF20 / CAP06	IBS001941	-	312.019		(2E, 6Z) - 2, 6- bis(3, 3- dichloroallylidene) cyclohexanone
MF20 / CAP06	IBS002761	70402-14-1	195.217		6- amino- 1H- phenalen- 1- one
MF20 / CAP06	IBS004204	-	263.355		3- (2- oxo- 2- ((1S, 2S, 4S) - 1, 7, 7- trimethylbicyclo [2.2.1] heptan- 2- yloxy) ethyl) - 1H- imidazol- 3- ium
MF20 / CAP06	IBS004487	-	492.547		N- ((tetrahydrofuran- 2- yl) methyl) - 2- (3- ((2, 4, 6- trioxo- 1- (thiophen- 2- ylmethyl) tetrahydropyrimidin- 5(6H) - ylidene) methyl) - 1H- indol- 1- yl) acetamide
MF20 / CAP06	IBS004820	-	342.369		2-[(3-phenyl-4,5-dihydro-1,2-oxazol-5-yl)methyl]-1,2-benzothiazol-3(2H)-one 1,1-dioxide
MF20 / CAP06	IBS005697	-	422.457		4- ethyl- N- (2- (4- methoxyphenylcarbamoyl) benzofuran- 3- yl) - 1, 2, 3- thiadiazole- 5- carboxamide
MF20 / CAP06	IBS005747	-	294.333		(E) - N- (3- (1H- imidazol- 1- yl) propyl) - 2- (2- aminothiazol- 4- yl) - 2- (hydroxyimino) acetamide
MF20 / CAP06	IBS006016		401.502		ethyl 2- (2- (thiophen- 2- yl) acetamido) - 4- p- tolylthiazol- 5- ylcarbamate
MF20 / CAP06	IBS006225	-	256.217		5- (4- (1H- tetrazol- 1- yl) phenyl) furan- 2- carboxylic acid
MF20 / CAP06	IBS006519	-	513.523		(E) - methyl 5- ((S, Z) - 4- benzamido- 3- (2- (3, 5- dinitrophenyl) hydrazono) dihydrothiophen- 2(3H) - ylidene) pentanoate
MF20 / CAP06	IBS006755	-	238.24		2- (2- (isopropylamino) - 1- nitrovinyl) benzene- 1, 4- diol
MF20 / CAP06	IBS006810	-	301.342		2- (1H- benzo [d] imidazol- 2- yl) - N- (4- fluorophenyl) hydrazinecarbothioamide
MF20 / CAP06	IBS007304	-	352.495		ethyl 5'- (3- allylthioureido) - 2, 3'- bithiophene- 4'- carboxylate
MF20 / CAP06	IBS007936	-	470.611		4- amino- N- (4- (dimethylamino) phenyl) - 2- (2- (4- (dimethylamino) phenylamino) - 2- oxoethylthio) thiazole- 5- carboxamide

1st screening	Product Number	CAS No.	Molecular Weight	Name	IUPAC
MF20 / CAP06	IBS008528	-	299.348		2- (4- hydroxyphenylamino) - 5- ((1- methyl- 1H- pyrrol- 2- yl) methylene) thiazol- 4(5H) - one
MF20 / CAP06	IBS008808	-	359.443		(E) - N- (2- (5, 6- dihydroimidazo [2, 1- b] thiazol- 3- yl) ethyl) - 3- (3, 4- dimethoxyphenyl) acrylamide
MF20 / CAP06	IBS009385	-	475.968		2-({[(5Z)-5-(2-chlorobenzylidene)-2,4-dioxo-1,3-thiazolidin-3-yl]acetyl}amino)-4,5,6,7-tetrahydro-1-benzothiophene-3-carboxamide
MF20 / CAP06	IBS009878	-	396.459		(2Z)-5,5-dimethyl-2-({[(4-methylphenyl)sulfonyl]oxy}imino)-5,6-dihydropyrrolo[2,1-a]isoquinolin-3(2H)-one
MF20 / CAP06	IBS010307	-	306.267		ethyl 5-[2-oxo-2-(2,3,4-trihydroxyphenyl)ethyl]furan-2-carboxylate
MF20 / CAP06	IBS010844	-	389.203		N'-[(E)-(5-bromo-2-hydroxyphenyl)methylidene]-3-(5-methylfuran-2-yl)-1H-pyrazole-5-carbohydrazide
MF20 / CAP06	IBS011137	-	543.615		ethyl 1-[(3-{2-[(2E)-2-(3-nitrobenzylidene)hydrazinyl]-1,3-thiazol-4-yl}phenyl)sulfonyl]piperidine-4-carboxylate
MF20 / CAP06	IBS011591	-	196.208		2-(pyridin-3-yl)-2H-benzotriazole
MF20 / CAP06	IBS012158	29917-12-2	129.114		3- hydroxyiminopentane- 2, 4- dione
MF20 / CAP06	IBS012297	-	184.113		(5Z,6Z)-N,N'-dihydroxy[1,2,5]oxadiazolo[3,4-b]pyrazine-5,6(4H,7H)-diimine
MF20 / CAP06	IBS012365	-	200.142		(2E) - N- (3, 4- difluorophenyl) - 2- hydroxyiminoacetamide
MF20 / CAP06	IBS012467	41153-83-7	215.004		6-bromo-2,1,3-benzoxadiazole 1-oxide
MF20 / CAP06	IBS012712	-	241.249		(6E)-N-hydroxy-5-methyl-7-phenyl[1,2,4]triazolo[1,5-a]pyrimidin-6(7H)-imine
MF20 / CAP06	IBS012882	-	254.241		(2E)-N-(dibenzo[b,d]furan-2-yl)-2-(hydroxyimino)ethanamide
MF20 / CAP06	IBS013808	23821-62-7	301.383		[2-phenyl-4-(thiophen-2-yl)-1,3-thiazol-5-yl]acetic acid
MF20 / CAP06	IBS013949	-	307.386		(1R,6S)-N-{[(4-fluorophenyl)carbamothioyl]amino}bicyclo[4.1.0]heptane-7-carboxamide
MF20 / CAP06	IBS015578	-	362.596		4,4,4-trichloro-3-(1H-imidazol-1-yl)-1-(4-nitrophenyl)butan-1-one
MF20 / CAP06	IBS017087	-	408.407		N'-[(Z)-(4-hydroxy-3-methoxy-5-nitrophenyl)methylidene]-2,3,4,9-tetrahydro-1H-carbazole-6-carbohydrazide
DS / TMDU Chemical Library	SIGMA-L3523	80224-16-4	887.040	[Trp4]-Kemptide (Leu-Arg-Arg-Trp-Ser-Leu-Gly)	2-[[2-[[2-[[2-[[2-[[2-[(2-amino-4-methylpentanoyl)amino]-5-(diaminomethylideneamino)pentanoyl]amino]-5-(diaminomethylideneamino)pentanoyl]amino]-3-(1H-indol-3-yl)propanoyl]amino]-3-hydroxypropanoyl]amino]-4-methylpentanoyl]amino]acetic acid
DS / TMDU Chemical Library	SIGMA-V6879	50-57-7	1056.218	[Lys8]-Vasopressin	N-[6-amino-1-[(2-amino-2-oxoethyl)amino]-1-oxohexan-2-yl]-1-[19-amino-7-(2-amino-2-oxoethyl)-10-(3-amino-3-oxopropyl)-13-benzyl-16-[(4-hydroxyphenyl)methyl]-6,9,12,15,18-pentaoxo-1,2-dithia-5,8,11,14,17-pentazacycloicosane-4-carbonyl]pyrrolidine-2-carboxamide
DS / TMDU Chemical Library	SIGMA-K1127	65189-71-1	771.908	Kemptide Acetate salt (for PKA) (Leu-Arg-Arg-Ala-Ser-Leu-Gly)	2-[[(2S)-2-[[(2S)-2-[[(2S)-2-[[(2S)-2-[[(2S)-2-[[(2S)-2-amino-4-methylpentanoyl]amino]-5-(diaminomethylideneamino)pentanoyl]amino]-5-(diaminomethylideneamino)pentanoyl]amino]propanoyl]amino]-3-hydroxypropanoyl]amino]-4-methylpentanoyl]amino]acetic acid

1st screening	Product Number	CAS No.	Molecular Weight	Name	IUPAC
DS / TMDU Chemical Library	SIGMA-A9202	51833-78-4	899.005	Angiotensin Fragment 1-7 acetate salt hydrate (Asp-Arg-Val-Tyr-Ile-His-Pro)	1-[2-[[2-[[2-[[2-[[2-[(2-amino-3-carboxypropanoyl)amino]-5-(diaminomethylideneamino)pentanoyl]amino]-3-methylbutanoyl]amino]-3-(4-hydroxyphenyl)propanoyl]amino]-3-methylpentanoyl]amino]-3-(1H-imidazol-5-yl)propanoyl]pyrrolidine-2-carboxylic acid
DS / TMDU Chemical Library	SIGMA-A2275	34273-10-4	912.047	[Sar1, Val5, Ala8]-Angiotensin II acetate salt hydrate (Saralasinum) (Sar-Arg-Val-Tyr-Val-His-Pro-Ala)	2-[[1-[2-[[2-[[2-[[2-[[5-(diaminomethylideneamino)-2-[[2-(methylamino)acetyl]amino]pentanoyl]amino]-3-methylbutanoyl]amino]-3-(4-hydroxyphenyl)propanoyl]amino]-3-methylbutanoyl]amino]-3-(1H-imidazol-5-yl)propanoyl]pyrrolidine-2-carbonyl]amino]propanoic acid
DS / TMDU Chemical Library	SIGMA-B1901	16875-11-9	904.023	Bradykinin Fragment 2-9 (Pro-Pro-Gly-Phe-Ser-Pro-Phe-Arg)	5-(diaminomethylideneamino)-2-[[2-[[1-[3-hydroxy-2-[[3-phenyl-2-[[2-[[1-(pyrrolidine-2-carbonyl)pyrrolidine-2-carbonyl]amino]acetyl]amino]propanoyl]amino]propanoyl]pyrrolidine-2-carbonyl]amino]-3-phenylpropanoyl]amino]pentanoic acid
DS / TMDU Chemical Library	Peptide Inst. #4028	100900-06-9	931.091	Angiotensin III, Human (Arg-Val-Tyr-Ile-His-Pro-Phe)	2-[[1-[2-[[2-[[2-[[2-[[2-amino-5-(diaminomethylideneamino)pentanoyl]amino]-3-methylbutanoyl]amino]-3-(4-hydroxyphenyl)propanoyl]amino]-3-methylpentanoyl]amino]-3-(1H-imidazol-5-yl)propanoyl]pyrrolidine-2-carbonyl]amino]-3-phenylpropanoic acid
DS / TMDU Chemical Library	SIGMA-L5387	51776-33-1	747.842	Luteinizing Hormone-Releasing Hormone Fragment 4-10 (LH-RH Fragment 4-10, Ser-Tyr-Gly-Leu-Arg-Pro-Gly-NH2)	1-[2-[[2-[[2-[[2-[(2-amino-3-hydroxypropanoyl)amino]-3-(4-hydroxyphenyl)propanoyl]amino]acetyl]amino]-4-methylpentanoyl]amino]-5-(diaminomethylideneamino)pentanoyl]-N-(2-amino-2-oxoethyl)pyrrolidine-2-carboxamide
DS / polyA.A.			786.791	Gln-Gln-Gln-Gln-Gln-Gln (6Q)	
DS / polyA.A.			977.990	His-His-His-His-His-His-His (7H)	
